# Population dynamics linked to glacial cycles in *Cercis chuniana* F. P. Metcalf (Fabaceae) endemic to the montane regions of subtropical China

**DOI:** 10.1111/eva.13301

**Published:** 2021-10-07

**Authors:** Wanzhen Liu, Jianguang Xie, Hui Zhou, Hanghui Kong, Gang Hao, Peter W. Fritsch, Wei Gong

**Affiliations:** ^1^ Guangdong Laboratory for Lingnan Modern Agriculture, & College of Life Sciences South China Agricultural University Guangzhou China; ^2^ Key Laboratory of Plant Resources Conservation and Sustainable Utilization, South China Botanical Garden Chinese Academy of Sciences Guangzhou China; ^3^ Center of Conservation Biology Core Botanical Gardens Chinese Academy of Sciences Guangzhou China; ^4^ Botanical Research Institute of Texas Fort Worth Texas USA

**Keywords:** *Cercis chuniana*, demographic modeling, geographical isolation, mountains, Pleistocene glacial cycles, secondary contact, subtropical China

## Abstract

The mountains of subtropical China are an excellent system for investigating the processes driving the geographical distribution of biodiversity and radiation of plant populations in response to Pleistocene climate fluctuations. How the major mountain ranges in subtropical China have affected the evolution of plant species in the subtropical evergreen broadleaved forest is an issue with long‐term concern. Here, we focused on *Cercis chuniana*, a woody species endemic to the southern mountain ranges in subtropical China, to elucidate its population dynamics. We used genotyping by sequencing (GBS) to investigate the spatial pattern of genetic variation among 11 populations. Geographical isolation was detected between the populations located in adjacent mountain ranges, thought to function as geographical barriers due to their complex physiography. Bayesian time estimation revealed that population divergence occurred in the middle Pleistocene, when populations in the Nanling Mts. separated from those to the east. The orientation and physiography of the mountain ranges of subtropical China appear to have contributed to the geographical pattern of genetic variation between the eastern and western populations of *C*. *chuniana*. Complex physiography plus long‐term stable ecological conditions across glacial cycles facilitated the demographic expansion in the Nanling Mts., from which contemporary migration began. The Nanling Mts. are thus considered as a suitable area for preserving population diversity and large population sizes of *C*. *chuniana* compared with other regions. As inferred by ecological niche modeling and coalescent simulations, secondary contact occurred during the warm Lushan–Tali Interglacial period, with intensified East Asia summer monsoon and continuous habitat available for occupation. Our data support the strong influence of both climatic history and topographic characteristics on the high regional phytodiversity of the subtropical evergreen broadleaved forest in subtropical China.

## 1 INTRODUCTION

High physiographical heterogeneity is suggested to prompt rapid diversification in montane habitats because of the increased ecological opportunities afforded by frequent episodes of geographical isolation (Colin & Ruth, 2006; Muellner‐Riehl, 2019; Simpson, 1964). Subtropical China comprises a geographically and climatically heterogeneous mid‐elevation montane region bordered by the Qinling Mountains–Huai River (~34°N) in the north, the tropical region (~22°N) in the south, the Qinghai–Tibetan Plateau in the west, and the coastline in the east (Wu, 1980; Wu et al., 1987). Many studies have identified the mountains of subtropical China as one of the main centers of phytodiversity and endemism in the country and globally, with much higher diversity than the other regions in the Northern Hemisphere (López‐Pujol et al., 2011; Qian et al., 2005). The high biodiversity of subtropical China is due in large part to the extreme physiographical heterogeneity of its mountain ranges (Fan et al., 2018; Xu et al., 2017; Yang et al., 2016). Generally oriented in north–south or northeast–southwest directions (Hou, 1983; Wang, 1992a, 1992b; Ying, 2001), these topographically diverse ranges have been suggested to serve as either geographical barriers or colonization corridors for various plant species (Gong et al., 2016; Tian et al., 2018; Xiong et al., 2019). The uniqueness of their local habitats has been attributed to complex topography correlated with longitudinal or steep elevational gradients (Qiu et al., 2013; Wang et al., 2012). The primary vegetation type of these mountains is subtropical evergreen broadleaved forest (STEBF), one of the largest continuous such forests in the world and well known for harboring ancient relictual elements of the north temperate biota (Qiu et al., 2011; Wang et al., 2012). Many of their plant species, predominantly endemics, exhibit high rates of local and rapid radiation (Hou et al., 2017; López‐Pujol et al., 2011) presumably arising within the last 5 million years, in line with both orogenic events and Pleistocene glacial cycles (Li et al., 1979; Liu et al., 2013; Shi et al., 1998; Wang et al., 2010). These characteristics make subtropical China an excellent system for investigating the processes driving the geographical distribution of biodiversity and radiation of plant populations in response to Pleistocene climate fluctuations.

Climatic oscillations associated with glacial cycles during the Pleistocene are also considered an important factor driving the distribution pattern of biodiversity and shaping the demographic history of populations, particularly in montane regions (Bueno de Mesquita et al., 2018; Hewitt, 2004; Li, Kong, et al., 2019; Li, Zhang, et al., 2019; Svenning et al., 2009). Although still under debate, considerable data are now available to support four glacial periods in eastern China (east to 105ºE) during the Pleistocene, that is, the Poyang, Dagu, Lushan and Tali glacials (Duan et al., 1980). In subtropical China, the degree of habitat connectivity is thought to have decreased during glacial periods, with vegetation belts lowering in latitude and contracted geographical ranges, allowing geographical isolation and genetic divergence to occur (Harrison et al., 2001; Shi et al., 2006). Multiple glacial refugia correlated with centers of genetic diversity have been identified in subtropical China, out of which subsequent localized or rapid range expansions have been inferred (Chen et al., 2012; Gong et al., 2008; Li et al., 2012; Qiu et al., 2011; Tian et al., 2015). Previous research has elucidated the scenarios involved with the evolutionary history of plant species thought to be affected by glacial and postglacial cycles in subtropical China (Gong et al., 2016; Liu et al., 2012; Tian et al., 2020). This research suggests that climate change is considered the main driver in triggering genetic differentiation and population divergence in subtropical China (Chen et al., 2020; Qiu et al., 2011; Wang et al., 2015, 2017). However, the extent to which topographic heterogeneity and the major mountain ranges of subtropical China may have affected the evolution of endemic species has been less studied. The studies that have been done on this issue suggest that topographic heterogeneity is as important as climate fluctuations in driving the evolution of species diversity in subtropical China (Li, Kong, et al., 2019; Li, Zhang, et al., 2019; Liu et al., 2014; Zhang et al., 2018; Zhu et al., 2019).

*Cercis chuniana* F.P. Metcalf (Fabaceae: Cercidoideae; Azani et al., 2017) is a small tree or shrub endemic to the STEBF of southern China. In comparison with its congeners, including *C*. *canadensis* L., *C*. *glabra* Pamp. and *C*. *siliquastrum* L., which have wide‐ranging distributions with large population sizes, *C*. *chuniana* has a relatively narrow geographical distribution. It occurs in the major mountain ranges in subtropical China, extending from the Wuyi and Eastern China Mountains westward to the Nanling Mountains. As with its congeners in China, it exhibits an adaptation to mesic environments by its acuminate leaf blade apex (Fritsch & Cruz, 2012; Fritsch et al., 2018; Isely, 1975; Wunderlin et al., 1981). Unique among *Cercis* species, it has an asymmetrical leaf blade (Chen et al., 2010; Metcalf, 1940), which makes it easily identifiable morphologically. The species is resolved near the base of the *Cercis* phylogenetic tree, and the species diversification time is estimated to be 2.40 Ma based on fossil‐calibrated divergence time analysis in *Cercis* (Fritsch et al., 2018; Liu et al., unpublished data, 2020). Therefore, we considered *C*. *chuniana* as a strong candidate for investigating the influence of both climatic history and topographic characteristics on the high regional phytodiversity of the subtropical evergreen broadleaved forest in southern China.

Genotyping by sequencing (GBS) is a streamlined workflow for generating reduced representation libraries for Illumina sequencing (Heffelfinger et al., 2014; Ilut et al., 2014; Melo et al., 2016) and has been widely used as a genomic approach for investigating genetic diversity and population structure (Chen et al., 2017; Metzker, 2010; Niu et al., 2019). Because it is based on genomic reduction with restriction enzymes, GBS does not require a reference genome to detect single nucleotide polymorphisms (SNPs). In combination with marker discovery and genotyping, GBS provides a rapid, high‐throughput, and cost‐effective tool for a genome‐wide analysis for nonmodel species (Andrews et al., 2016; Davey et al., 2011; Scheben et al., 2017). Here, we used GBS and collected genome‐wide SNPs for population genetic analyses of *C*. *chuniana*. We aimed to (1) investigate genetic diversity and population structure of the species, (2) elucidate its demographic history, and (3) use the data to test the relative influence of topographic heterogeneity versus Pleistocene climatic fluctuations in driving population diversification and geographical distribution within the STEBF in subtropical China.

## 2 MATERIALS AND METHODS

### 2.1 Population sampling

**FIGURE 1:**
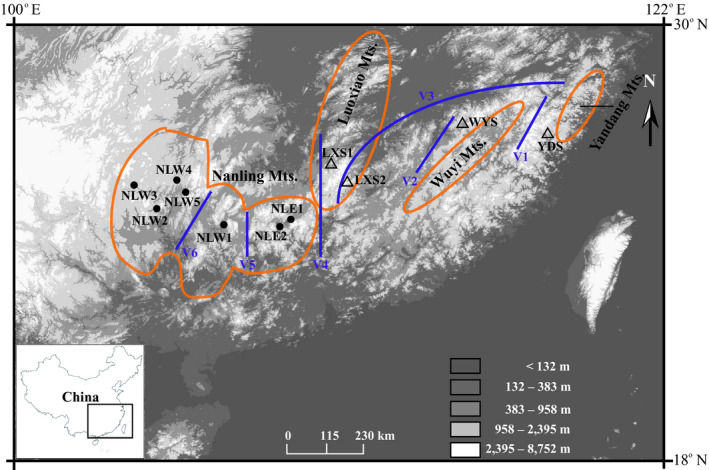
Map of subtropical China, showing the sampling locations of *Cercis chuniana* populations used in this study. The mountain ranges involved in the study are shown in orange frames. Populations sampled in the Nanling Mts. are indicated with black circles; populations in the east are indicated with triangles. Vicariance events detected with S‐DIVA are shown with blue lines. The key at bottom right indicates elevational ranges. Latitudes and longitudes are shown on the right side and top side, respectively. Additional accession information is given in Table 1

We collected 11 populations and 112 individuals of *C*. *chuniana* from throughout the current geographical distribution of the species (Figure 1, Table 1). Anywhere from one to five populations were collected from each of the mountain ranges in subtropical China. The sampled populations are located in the southern Yandang Mts. (YDS), the northern Wuyi Mts. (WYS), the southern Luoxiao Mts. (LXS1 and LXS2), the eastern Nanling Mts. (NLE1 and NLE2), and the western Nanling Mts. (NLW1 through NLW5; Figure 1, Table 1). Some population sizes are very small with limited numbers of living individuals because of the destruction of habitat. Therefore, less than ten individuals were collected in five small populations, including YDS, WYS, LXS1, NLW2, and NLW5. We also collected 20 individuals from one population of *C*. *chingii* Chun located in Chichengshan, Zhejiang Province (CCS), which were used as the outgroup. The reason that we chose *C*. *chingii* as the outgroup is mainly based on the ML tree constructed for *Cercis* based on GBS data, which shows that *C*. *chuniana* is at the basal branch followed by *C*. *chingii*. Therefore, we chose *C*. *chingii* as the outgroup as the other congeneric species are phylogenetically much most distant. Additionally, we tested the result of the ML tree by randomly choosing one individual of *C*. *chingii*, which did not change the final topology.

**TABLE 1:** Accession information and genetic diversity parameters of observed heterozygosity (Ho) and expected heterozygosity (He) for the 11 *Cercis chuniana* populations and the outgroup *C*. *chingii*

No.	Population	Location	Mountain range	*N*	Latitude	Longitude	Elevation range (m)	He/Ho	Pi
	*C. chuniana*								
1	YDS	Tonglingshan, Wenzhou, Zhejiang	Southern Yandang Mts.	8	27.82°N	119.85°E	264‒727	0.32/0.31	15.90
2	WYS	Wuyi Mts., Nanping, Fujian	Western Wuyi Mts.	9	27.94°N	117.77°E	460‒712	0.34/0.28	8.17
3	LXS1	Jinggangshan, Ji'an, Jiangxi	Southern Luoxiao Mts.	9	26.78°N	113.90°E	264‒509	0.32/0.30	2.44
4	LXS2	Wuzhifeng, Ganzhou, Jiangxi	Southern Luoxiao Mts.	14	26.00°N	114.15°E	369‒722	0.31/0.19	5.77
5	NLE1	Mangshan Nature Reserve, Chenzhou, Hunan	Eastern Nanling Mts.	12	24.98°N	112.89°E	716	0.32/0.21	3.53
6	NLE2	Dadongshan Nature Reserve, Qingyuan, Guangdong	Eastern Nanling Mts.	10	24.92°N	112.72°E	774	0.32/0.32	1.36
7	NLW1	Yindianshan Nature Reserve, Guilin, Guangxi	Western Nanling Mts.	11	24.91°N	110.96°E	700	0.33/0.34	1.75
8	NLW2	Tianpingshan, Huaping Nature Reserve, Guilin, Guangxi	Western Nanling Mts.	5	25.61°N	109.90°E	852	0.38/0.33	8.60
9	NLW3	Dupoxiang, Huaihua, Hunan	Western Nanling Mts.	13	26.07°N	109.47°E	612‒657	0.32/0.30	1.54
10	NLW4	Nanshan Log Yard, Shaoyang, Hunan	Western Nanling Mts.	14	26.18°N	110.19°E	611‒703	0.32/0.30	1.03
11	NLW5	Mao'ershan National Nature Reserve, Guilin, Guangxi	Western Nanling Mts.	7	25.89°N	110.38°E	700‒852	0.35/0.25	4.48
	*C. chingii*								
12	CCS	Chichenshan, Tiantai, Taizhou, Zhejiang	Xianxia Mts.	20	29.17°N	121.03°E	167‒236		

### 2.2 Ecological niche modeling

We used ecological niche modeling (ENM; Soberón & Peterson, 2005) to characterize the spatial distribution of suitable conditions for *C*. *chuniana* and locate potential distributional areas in conjunction with historical biological inferences. We based the analysis on high‐resolution paleoclimate data inferred for the Last Interglacial (LIG, 0.14~0.12 Ma), Last Glacial Maximum (LGM, ≈ 0.02 Ma), Middle Holocene (MH, ≈ 0.006 Ma), and current. Bioclimatic variables were downloaded from the WorldClim database (http://worldclim.org/download; Fick & Hijmans, 2017) for the four different stages with 2.5‐minute spatial resolution. The LIG, LGM, and MH data were obtained from circulation model simulation of the Community Climate System Model (CCSM; Collins et al., 2006), which provides downscaled high‐resolution estimates of the climate parameters (Hijmans et al., 2005). We used the maximum entropy modeling method with Maxent v3.3.2 (Phillips et al., 2006). Herbarium specimen records of *C*. *chuniana* from nine herbaria (A, IBEC, IBK, IBSC, KUN, LBG, NMNH, PE, and SCFI) and our sample collection locations were used to determine the locations of populations considered to occur at present. The analysis pipelines and parameter settings, including the occurrence points, current/past bioclimatic variables and the convergence threshold and maximum number of iterations, were all as in Dai et al. (2011) and Gong et al. (2016). Model accuracy was assessed by evaluating the area under the curve (AUC) of the receiver operating characteristic (ROC) plot (Phillips et al., 2006), where scores higher than 0.70 were considered to show good model performance (Fielding & Bell, 1997). This approach is thus conservative, identifying the minimum predicted area possible while maintaining zero omission error in the training dataset (Pearson et al., 2007). We added a layer of GIS‐based vegetation map for comparison in each period of LIG, MH, LGM and current (Allen et al., 2020; Ray & Adams, 2001). The most influential climate factors were also compared, including precipitation and temperature in each month or on average.

### 2.3 DNA extraction, genotyping by sequencing (GBS), SNP calling, and quality filtering

Fresh leaves of *C*. *chuniana* and *C*. *chingii* were sampled and placed into centrifuge tubes, which were instantly immersed in liquid nitrogen and stored at −80℃. Leaf tissue was ground in tubes with glass beads with the tissue homogenizer TissueLyser‐96 (Shanghai Jingxin Industrial Development Co., Ltd). Total genomic DNA was extracted with the modified cetyl trimethyl ammonium bromide (CTAB) method (Doyle & Doyle, 1986). DNA concentration was quantified with a NanoDrop spectrophotometer (Thermo Scientific), and a final DNA concentration of >30 ng/µl was used.

The genomic DNA was digested with a combination of *Mse*I and *Nla*III enzymes. Subsequent ligation to barcodes after multiplex amplification was constructed, and the desired fragments were selected for GBS library construction in Novogene Co., Ltd. The Illumina HiSeq sequencing platform (Illumina) was used for paired‐end (PE) 150 sequencing. Further analyses and DNA library assembly were performed to remove low‐quality reads. Reads in fastq format were assembled by using STACKS v2.2 (Catchen et al., 2013) with one individual of *Cercis glabra* as reference and up to six base mismatches allowed. BWA v0.7.8 (Li & Durbin, 2009) was used for sequence mapping and sorting with the following settings: mem ‐t 4 ‐k 32 ‐M. The alignment files were converted to bam files with SAMtools v1.3.1 (Li et al., 2009). We used 132 individuals for SNP calling with Stacks. For population analysis, we extracted SNPs with a minor allele frequency (MAF) of at least 0.05 and a genotyping rate of at least 80% of individuals within populations. We also specified a maximum observed heterozygosity of 50% and a minimum number of five populations per locus.

### 2.4 Phylogenetic analysis and divergence time estimation

Using the SNPs extracted from the GBS dataset, we employed maximum likelihood (ML) to reconstruct phylogenetic relationships among the 11 populations of *C*. *chuniana*. We used *C*. *chingii* to root the trees based on FastStructure analysis showing a close relationship between *C*. *chuniana* and *C*. *chingii* (Figure 4) and Mega analysis showing no remarkable differences in genetic distances between *C*. *chuniana* and other species. Therefore, we consider that *C*. *chingii* is sufficiently close to the ingroup for the purpose of rooting the tree in the ML analysis. Analyses were performed on the high‐performance computer cluster available in the CIPRES Science Gateway 3.3 (www.phylo.org; Miller et al., 2015). The ML analyses were performed simultaneously with 1000 ML bootstrap pseudoreplicates in RAxML v8 (Alexandros, 2014). The model of nucleotide substitution was selected with the Akaike information criteria (AIC; Akaike, 1974) in PhyML‐SMS (http://www.atgc‐montpellier.fr/phymL/; Lefort et al., 2017).

We used fossil calibrations for the estimation of divergence time in *Cercis*. The fossil age of *Cercis* was originally estimated as 34 Ma (Lavin et al., 2005), but recently updated to 36 Ma (Fritsch et al., 2018). We conducted divergence time estimation based on all *Cercis* species using the fossil calibration of 36 Ma at the crown node of the genus. The result indicated that the root age for *Cercis* is 33.53 Ma and the crown age for *C*. *chuniana* is 2.39 (Figure S2; Liu et al., unpublished data, 2020), the latter of which was used for further analysis of time divergence for all the *C*. *chuniana* populations. Therefore, to estimate the divergence time within *C*. *chuniana*, we used BEAST v2.4.7 (Bouckaert et al., 2014) and applied the age of 2.4 Ma as the secondary calibration point with a normal prior distribution and standard deviation of 0.2 Ma, which covered the 95% HPD range. The divergence time analyses were conducted with the GTR + G + I model and four rate categories, a Coalescent Constant Population prior, and the Strict Clock setting with uncorrelated and log‐normal‐distributed rate variation across the branches. We ran the MCMC simulations in BEAST for 10 million generations with parameters sampled every 1000th generation. We used Tracer v1.6 (Rambaut et al., 2014) to assess convergence and to check that the effective sample size (ESS) was >200 for each parameter. We discarded the first 10% of trees as burn‐in with the mean node heights option, and then generated the maximum clade credibility (MCC) chronogram from the remaining trees with nodal mean heights and 95% confidence time intervals with TreeAnnotator v2.4.7 (Bouckaert et al., 2014) in BEAST. The final trees were edited with FigTree v.1.4.3 (http://tree.bio.ed.as.uk/software/figtree/; Rambaut, 2014).

### 2.5 Genetic diversity, population assignment, and admixture

The number of alleles and allele frequencies for the selected SNPs were calculated with vcftools 0.1.16 (Danecek et al., 2011). To measure genetic diversity, we estimated expected heterozygosity (He) and observed heterozygosity (Ho). We used Arlequin v3.5 (Excoffier & Lischer, 2010) to estimate genetic differentiation by calculating pairwise values of differences among populations (Fst). To compare molecular diversity between and within populations, we used analysis of molecular variance (AMOVA) and a hierarchical analysis of subdivision (Excoffier et al., 1992; Weir, 1996; Weir & Cockerham, 1984). Altogether, seven groups were defined on the basis of FastStructure analysis.

We estimated population genetic structure with a Bayesian Markov chain Monte Carlo (MCMC) model implemented in FastStructure v1.0 (Raj et al., 2014). We used the default setting with 10‐fold cross‐validation on the 112 individuals of *C*. *chuniana*, testing for subpopulations (*K*) ranging from 1 to 11. The python script Choose K in FastStructure was used to choose the optimal *K*, that is, the value that maximizes the marginal likelihood. Results were graphically represented and edited with Adobe Illustrator. We performed principal component analysis (PCA) using the PCA function in SNPRelate (Zheng et al., 2012) and visualized the results using the scripts of Tanya Lama (https://github.com/ECOtlama/SNPRelate.git) in the R package.

### 2.6 Inference of demographic history

For ancestral area reconstruction, we used seven groups of *C*. *chuniana* for S‐DIVA (statistical dispersal–vicariance analysis) analysis implemented in RASP v3.2 (Ronquist, 1997; Yu et al., 2015). The analysis was based on the BEAST MCMC trees and the maximum clade credibility tree derived from the Bayesian analysis with BEAST and TreeAnnotator (Matuszak et al., 2016). With this method, the frequencies of an ancestral area at a node in the ancestral reconstructions are averaged over all trees. Dispersal or vicariance events were also detected with S‐DIVA.

We applied coalescent simulations with the program fastsimcoal2 (FSC2; Excoffier et al., 2013) to provide model evidence of divergence, secondary contact, bottleneck effects, and demographic expansion. The populations in the Nanling Mts. (NL), which formed a monophyletic group and were distinct geographically, were delimited as one group, and the remaining populations as another, that is, the eastern populations (ES). We used easySFS (https://github.com/isaacovercast/easySFS) to transform SNPs into a folded site frequency spectrum (SFS) based on the construction of 10 demographic models with the two groups (Figure S3). The models are as follows: without isolation (NIS), isolation only (IS), isolation followed by migration (MIG), bottleneck effect (BOT) or secondary contact (SEC). Models including ancient (AMIG) or recent migration (RMIG), bidirectional or one‐way migration, and demographic expansion (EXP) were also applied. In each model, NL or ES were alternatively used as the split source that was subjected to each scenario. We estimated effective population size (*Ne*), time (*T*) and migration rates in individual migrants per generation (*M_NL_*_‐_ *_ES_ and M_ES_*_‐_ *_NL_*) for the two groups in each model from posterior distributions. To scale parameter estimates into real values, we calculated the substitution/site/generation mutation rate based on phylogenetic analysis and divergence time estimate of *Cercis* (Liu et al., unpublished data, 2020), because the genomic mutation rate has not been calculated for this genus. The clock rate was firstly estimated as 2.32 × 10^−8^ substitutions/site/year for nine species and 241 individuals of *Cercis* with Tracer v1.6 (Rambaut et al., 2014). With a generation time of five years as based on congeners (Aldworth, 1998; Chen & Mao, 1999), the mutation rate was calculated as 1.16 × 10^−7^ substitutions/site/generation. As compared to some other plants such as *Arabidopsis*, *Prunus*, and *Silene* that show ~7 × 10^−9^ substitutions/site/year, the substitution rate for *C*. *chuniana* appears to be faster. However, the substitution/mutation rates vary in a wide range among different plant species and are strongly associated with the life history traits and generation time (Smith & Donoghue, 2008). We ran 100 replicate FSC2 analyses under each model with 10,000 simulations for optimal parameters and composite likelihood estimation. All 10 demographic models were compared (Figure S3, Tables S2–S4). The composite likelihood of arbitrarily complex demographic models under the given SFS was calculated by using best‐fit models based on the Akaike information criterion (AIC). The models with the lowest AIC were chosen as the best fit of the data (Akaike, 1974).

## 3 RESULTS

### 3.1 Ecological niche modeling

**FIGURE 2:**
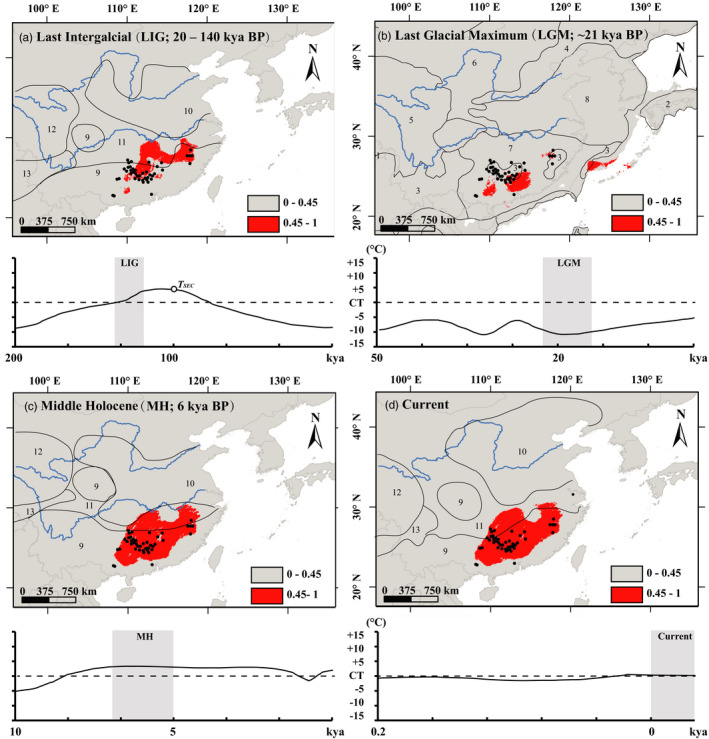
Potentially suitable areas for *Cercis chuniana* predicted by ecological niche modeling (ENM) and corresponding variation in temperature for four different periods of LIG (a), LGM (b), MH (c), and Current (d). Suitable and unsuitable habitats are indicated in red and gray, respectively, where red represents the habitat suitability (occurrence probability) higher than 44.93%. Each map is shown in comparison with a layer of GIS‐based vegetation map for each period. Numbers 1‒13 represent different vegetation types: 1, tropical thorn scrub and scrub woodland; 2, open boreal woodland; 3, semi‐arid temperate woodland or scrub; 4, steppe‐tundra; 5, polar and alpine desert; 6, temperate desert; 7, forest steppe; 8, dry steppe; 9, temperate broadleaved evergreen forest; 10, warm temperate woodland; 11, temperate mixed forest; 12, shrub tundra, and 13, boreal evergreen coniferous forest. Gray boxes enclose the temperatures for that time interval. The *y*‐axis shows the temperatures compared with the current one (CT). The temperature during secondary contact (*T*_SEC_) is indicated in LIG (a). The most influential factors are listed in Table S1

Evaluation of model performance based on both training and test sample data indicated that the models had high predictive power (AUC = 0.9976 and 0.9966, respectively). Results yielded a continuous geographical distribution of *C*. *chuniana* across several mountain ranges in subtropical China during the Last Interglacial (LIG) period, under higher temperature than current (Figure 2a). In contrast, during the Last Glacial Maximum (LGM) the geographical distribution contracted into three fragmented areas, when the temperature was ~5–10℃ lower than current (Figure 2b). We compared these data with a vegetation map of the LGM (http://intarch.ac.uk/journal/issue11/2/map/download_page_js.htm), finding that the three fragmented distribution areas were located mainly inside forest steppe (number 7) and partially in semi‐arid temperate woodland or scrub (number 3; Ray & Adams, 2001). The geographical distribution of *C*. *chuniana* was inferred to have expanded widely during the Middle Holocene (MH), occupying most of the Chinese subtropical region. No changes in the geographical distribution were evident between MH and current. Precipitation in April, May, and June was revealed to be the most influential climate factor for the suitable distribution range of *C*. *chuniana* (Table S1).

### 3.2 Characterization and distribution of SNPs

GBS produced 200 Gb clean reads after filtration. High‐quality tags were identified from 10,761,958 Gb‐PE reads. The sequence data were high quality with Q20 ≥ 92.23% and Q30 ≥ 85.00%. The mean G + C content was 37.84%. We detected 61,748 SNPs for *C*. *chuniana* with *C*. *chingii* as outgroup, among which 32,890 SNPs agreed with the SNP extraction criteria. The data have been deposited in Figshare (https://doi.org/10.6084/m9.figshare.15283395).

### 3.3 Phylogenetic relationships and divergence times

**FIGURE 3:**
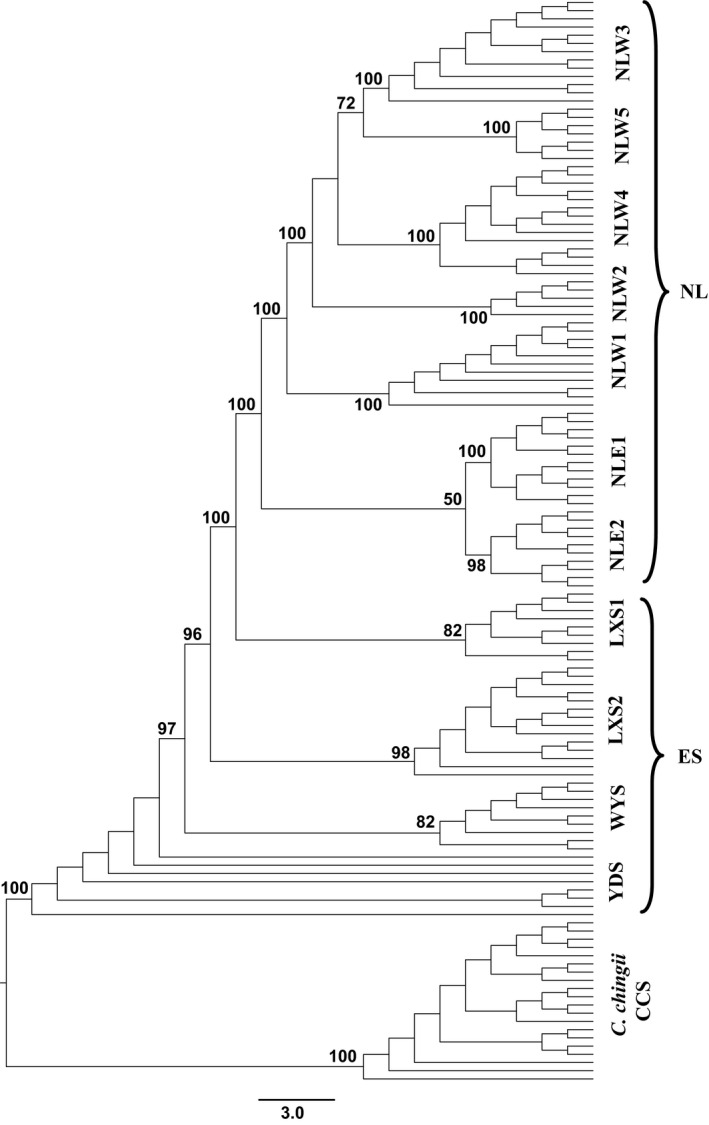
Phylogenetic tree of *Cercis chuniana* populations based on maximum‐likelihood (ML) analysis. Bootstrap percentages (>50) in the ML tree are indicated above the branches. NL refers to the populations in Nanling Mts., whereas ES refers to the populations in the east. All other abbreviations are population abbreviations from Table 1. *Cercis chingii* was used as the outgroup

**FIGURE 4:**
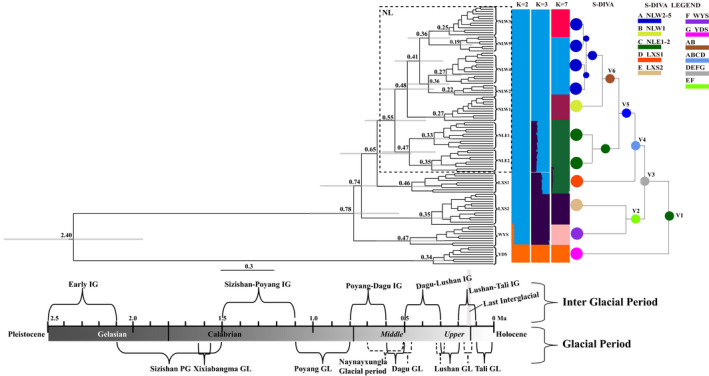
Chronogram of the Bayesian tree for divergence time estimates, population structural clustering and ancestral area reconstruction. Branch lengths were transformed via Markov chain Monte Carlo (MCMC) simulations in the Bayesian time estimation. The light gray bars indicate 95% confidence intervals. The key glacial and interglacial periods are indicated by the braces, with GL standing for glacial period, IG for interglacial period, and PG for periglacial period. Individuals assigned to different clusters in FastStructure are shown in corresponding colors with *K* = 2, 3, and 7, with 7 as the optimal value. In the S‐DIVA analysis, color legends indicate different geographical regions and ancestral areas. The individuals from the same population are represented in one colored circle. Vicariance events (V1–V6) with high probabilities (*p* ≥ 0.70) are shown for nodes. Populations in the Nanling Mts. are distinguished within the dotted line frame. Timescale bar is shown at the bottom. The population abbreviations are from Table 1

The phylogenetic analysis yielded monophyly for most populations with mostly high bootstrap values, except YDS (Figure 3 and Figure S1). YDS was revealed to be positioned at the first‐diverging branch, followed by the populations WYS, LXS2, and LXS1. The populations in the Nanling Mts. formed a monophyletic group, with NLE2 and NLE1 in the eastern Nanling Mts. forming a clade separate from the others in the western Nanling Mts. The time of origin for *C*. *chuniana* was estimated as 2.39 (95% HPD = 1.97–2.74) Ma during the early Pleistocene (Figure S2). YDS was first divergent from the remaining populations, followed by WYS diverging from the rest ca. 0.78 Ma during the end of the Poyang Interglacial period. The divergence occurring between LXS2 and the remaining populations was estimated as ca. 0.74 Ma, and the divergence between LSX1 and the populations in the Nanling Mts. as ca. 0.65 Ma. Both divergence times arose within the third glacial period in China in the Middle Pleistocene, although the exact glacial and interglacial time ranges are still under debate (Figure 4). Within the Nanling Mts., the eastern NLE1/NLE2 populations diverged from the western NLW1‒NLW5 populations ca. 0.55 Ma, and NLW1 from the rest of the western populations ca. 0.48 Ma, both during the Dagu Glacial period (Figure 4). Population diversification within the Nanling Mts. ranges from 0.19 to 0.27 Ma in the western populations and from 0.33 to 0.35 Ma in the eastern populations, the former during the Lushan Glacial period and the latter during the Dagu–Lushan Interglacial period. Population diversifications in the east (YDS, WYS, LXS1 and LXS2) range from 0.34 to 0.47 Ma, spanning the Dagu–Lushan Glacial and Interglacial periods (Figure 4).

### 3.4 Genetic diversity and differentiation

**FIGURE 5:**
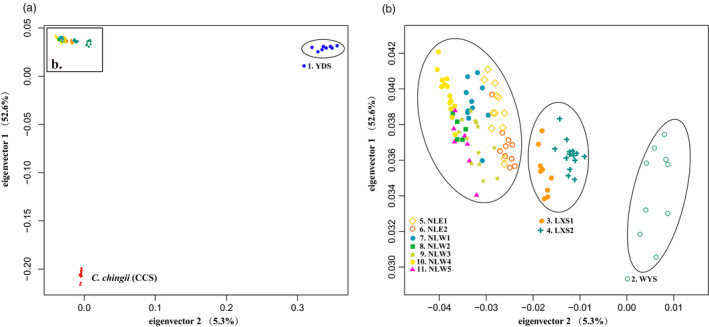
Principal component analysis (PCA) of *Cercis chuniana* populations. Different colors and shapes refer to each of the 11 populations of *C*. *chuniana* and one outgroup population of *C*. *chingii*

The highest He was detected in NLW2 (0.38) followed by NLW5 (0.35); the lowest was detected in LXS2 (0.31; Table 1). The highest H_0_ was detected in NLW1 (0.34) followed by NLW2 (0.33); the lowest was detected in LXS2 (0.19). On average, He and Ho in the Nanling Mts. (He = 0.33; Ho = 0.29) were comparable to those of the other populations in the east (He = 0.32; Ho = 0.27). In the FastStructure analysis, YDS separated from the remaining populations when *K* = 2. When *K* = 3, WYS and LXS2 clustered as one group and this group was separated from the remaining groups. Seven subpopulations (*K* = 7) were determined as the optimal clustering for *C*. *chuniana* (Figure 4). PCA results showed similar groupings except that LXS1 and LXS2 clustered together, and were distinct from WYS and from the Nanling Mts. populations (Figure 5). Considering the FastStructure, PCA, and phylogenetic results together with the geographical locations of populations, we ultimately circumscribed seven groups of *C*. *chuniana* populations for further analyses: YDS, WYS, LXS1, LXS2, [NLE1 + NLE2], NLW1, and [NLW2 through NLW5]. Analysis of the molecular variance based on the GBS data indicated significant genetic differentiation among populations (Fst = 0.99, *p* = 0.00), of which the variation among the seven groups accounted for 96.28% of the total variation (Table 2).

**TABLE 2:** Analysis of molecular variance (AMOVA) results for global Fst statistics of *Cercis chuniana*

Source of variation	*df*	Sum of squares	Variance components	Percentage of variation
Among groups	6	3367.488	37.06608	96.28%
Among populations within groups	4	36.908	0.89979**	2.34%
Within populations	101	53.747	0.53215**	1.38%
Total	111	3458.143	38.49802	
Fixation indices	Fsc: 0.6284**	Fst: 0.9862**	Fct: 0.9628	

Abbreviations: *df*, degrees of freedom; Fct, difference among groups; Fsc, differences among populations within groups; Fst, differences among all populations.

**p < 0.001.

### 3.5 Geographical isolation, secondary contact and demographic expansion

Six vicariance events (V1‒V6) among the geographical regions were inferred from the S‐DIVA analysis (Figure 1). V1 is between YDS and the rest of the populations. V2 is between WYS and LXS2, located in the western Wuyi Mts. and southern Luoxiao Mts., respectively. V3 is between WYS/LXS2 and the rest of the populations, including LXS1 and the populations in the Nanling Mts. V4 is between LXS1 and the rest of the populations. V5 is between the eastern and western Nanling Mts., separating [NLE1 + NLE2] and [NLW1 through NLW5], whereas V6 is between NLW1 and [NLW2 through NLW5]. Across the six vicariance events, the eastern populations diverged from the rest of the species first, and the western populations later.

**FIGURE 6:**
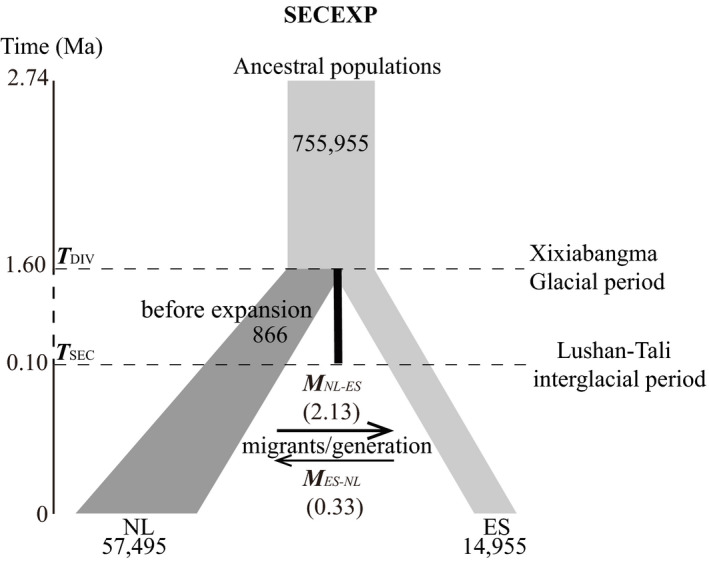
Schematic representation of the best‐fit demographic model investigated in our study. Model parameters correspond to those in Tables S2 and S3, respectively. The left vertical line shows time (Ma), which progresses from top to bottom (current time). Time of divergence (*T*_DIV_) and secondary contact (*T*_SEC_) are indicated as dashed lines, with the corresponding glacial or interglacial periods shown on the right. Vertical black bar represents a period of isolation of lineages before migration initiates at the secondary contact. The spanning time of this period is shown in dashed line on the left vertical timeline, which is shortened for visual purposes. The migration directions and corresponding rates (*M*_ES_) in individual migrants per generation are shown between NL and ES. Demographic expansion is indicated by the increasing areas of the gray ladder shapes. The current effective population sizes are shown at the bottom after the split between the Nanling Mts. (NL) and the eastern mountains (ES)

The best‐fit model for the demographic analysis with FSC2 is SECEXP, indicating isolation followed by secondary contact (SEC) and demographic expansion (EXP; Figure 6 and Figure S3, Tables S2–S4). In combination with the time tree given by BEAST, the timescale of 548,000 generations (2.74 Ma) was confirmed by the program fastsimcoal2 from the lowest AIC value. Based on the mutation rate, we converted the genome‐wide estimates of nucleotide diversity into effective population sizes. Nucleotide diversity per population is listed in Table 1. The current effective population sizes of the Nanling Mts. (NL) and eastern regions (ES) are Ne_NL_ = 57,495 and Ne_ES_ = 14,955, respectively. From the current effective population size, the ancestral effective population size was calculated as Ne_ANC_ = 755,955 (Figure 6, Table S3). Using the ancestral effective population size, we converted the divergence time between NL and ES into the number of generation times, *T*_DIV_ = 319,472 generations ago, that is, about 1.6 Ma. Secondary contact (SEC) was estimated at ca. *T*_SEC_ = 0.10 Ma. This date is within the Lushan–Tali Interglacial period in China (Duan et al., 1980; Zhu et al., 2004), when temperature increased and was ca. 5℃ higher than at present (Figure 2). The ancestral effective population size of NL was estimated to be much smaller (*N*_e‐pre‐exp_ = 866) than at present. In contrast, the ES population sizes remained more or less constant (Figure 6). The migration rate *M*_NL‐ES_ (2.14) was much higher than *M*_ES‐NL_ (0.33), with migration occurring after NL and ES divergence.

## 4 DISCUSSION

### 4.1 Geographical isolation associated with Pleistocene climatic oscillations and mountain ranges

As based on ML analysis (Figures 1, 3), populations of *C*. *chuniana* are mostly monophyletic and closely aligned with geographical regions except YDS suggesting that they evolved mostly via local diversification. This is thought to occur especially when geographical isolation plays a dominant role (Harrington et al., 2018; Hughes, 2017; Hughes & Atchison, 2015; Kadereit, 2017; Nevado et al., 2018; Xing & Ree, 2017). Analysis of the molecular variance with significantly high population divergence (Fst = 0.99, *p* = 0.00) also indicates low inter‐population gene flow (Table 2). Mountain ranges sometimes are considered as poorly conducive for facilitating long‐distance dispersal, thus contributing to limited gene flow and geographical isolation (Oyama et al., 2018). In our study, isolation between YDS and WYS (V1) was attributed to the Wuyi Mts. acting as geographical barrier to separate the populations from each other (Figures 1, 4). The rise of the Wuyi Mts. during the early Pleistocene is thought to have caused geographical isolation and genetic divergence for many species in subtropical China (Liu, 1984; Yan et al., 2013). Notably, the central Luoxiao Mts., with a north–south orientation, are assumed to have served as a geographical barrier particularly for east–west colonization. This appears to apply to LXS1 and LXS2 in the Luoxiao Mts., which are currently isolated from each of their eastern or western populations (V2 and V3; Figures 1, 4). We infer that the geographical isolation between the populations of the Nanling Mts. and those to the east (V4) has arisen through the lack of geographical corridors. Vicariance events also exist between the western and eastern (V5) and the middle and northwest (V6) populations within the Nanling Mts. The Nanling Mts. present a general north–south orientation, which we infer as disadvantageous for east–west colonization, thus contributing to vicariance involving V5. Unlike the populations NLW2~5, NLW1 is isolated on one ridge of the Nanling Mts. and geographically distant from the remaining populations, thus resulting in the vicariance involving V6. Therefore, the geographical barriers formed by the associated mountain ranges including the Wuyi, Luoxiao, and Nanling Mts. have directly limited long‐distance colonization and are considered a major factor contributing to the historical isolation of *C*. *chuniana* populations (Jiang et al., 2019; Li, Kong, et al., 2019; Li, Zhang, et al., 2019; Yang et al., 2019). Similar patterns have been found in many other plant species with a wide distribution range in subtropical China, such as *Machilus pauhoi* (Zhu et al., 2017), *Loropetalum chinense* (Gong et al., 2016), and *Liriodendron chinense* (Shen et al., 2019).

Our study suggests that population divergence of *C*. *chuniana* occurred in the Pleistocene and has been affected by the glacial cycles. These cycles periodically changed suitable habitat and are thought to have promoted range contraction and expansion coupled with geographical isolation (Knowles, 2001; Qu et al., 2011). Based on Bayesian estimation, the time of divergence (0.65 Ma) between the populations in the Nanling Mts. and those of the east coincides with the third (last) glacial period in China in the Middle Pleistocene (Figure 4). The time may fall in the Naynayxungla Glacial period (0.5–0.7 Ma; Zheng et al., 2002; Zhou & Li, 1998) or Poyang–Dagu Interglacial period (0.6–0.8 Ma; Duan et al., 1980). Although the precise time for the glaciations is under debate, it is at least clear that primarily the third (last) glaciation drove the genetic divergence between populations in the Nanling Mts. and those to the east, and shaped the geographical patterns of genetic variation. The estimated divergence time of the best‐fit model in FSC2 is older, that is, 1.60 Ma (Figure 6), which overlaps with the earliest known Quaternary glacial of the Xixiabangma Glacial period ca. 1.6 Ma (Wan et al., 2016), or the Sizishan Periglacial period (1.5–2.1 Ma; Duan et al., 1980), when the temperature was 10℃ lower than at present. The discrepancy between the results of Bayesian time estimation and FSC2 may be partially attributed to the wider time range under the log‐uniform setting in FSC2. The secondary calibration used in BEAST is thought to generate smaller time estimates (Foster et al., 2017; Kong, Condamine, et al., 2017; Kong, Zhang, et al., 2017). The climate during glacial periods tended to be dry and cool, which would favor the populations shifting to lower elevations or latitudes with contracted distribution ranges due to the reduced subtropical evergreen broadleaved forest during the glacial period. The glacial period in the Middle Pleistocene has been shown to have driven spruce fir forests to lowlands in northern China (Liu, 1988). In our study, the geographical distribution of *C*. *chuniana* in subtropical China is also associated with the Pleistocene glacial cycles (Figure 4). The Dagu Glacial period (0.5–0.6 Ma; Duan et al., 1980) primarily affected the population divergence between the east and west, whereas the Dagu–Lushan Interglacial period (0.3–0.5 Ma; Duan et al., 1980) and Lushan Glacial period (0.2–0.3 Ma; Duan et al., 1980) primarily affected population diversification. The dominant role for Pleistocene glacial cycles affecting the geographical distribution of populations is also apparent in ecological niche modeling (ENM), where several isolated glacial refugia were identified during the LGM, although the climatic conditions may not be analogous to those of other glacial cycles.

### 4.2 Genetic divergence between eastern and western populations

The population divergence in the eastern portion of the geographical range of *C*. *chuniana* is estimated to be older (0.47‒0.34 Ma) and with smaller population sizes than within the Nanling Mts., where more recent and rapid population diversification occurred (0.35‒0.19 Ma) with larger population sizes (Figure 4). The phylogenetic analysis also revealed that populations of the Nanling Mts. formed a monophyletic group and were distinctly separated from the eastern populations. This pattern agrees with the general pattern of genetic divergence observed between eastern and western China in other plant species with wide distributions (Chen et al., 2018; Gong et al., 2008; Ha et al., 2018; Hohmann et al., 2018; Lu et al., 2018; Qiu et al., 2009). One main factor contributing to the differences in population divergence time and level of diversification between the east and west is likely to be the different orientations of mountain ranges (Chen et al., 2018). The southwest–northeast orientation of the Wuyi Mts. and East China Mts. is thought to present geographical barriers that blocked southward migrations in times of cooler climate or northward postglacial population expansion, which is disadvantageous for increasing population size and diversification, and may have contributed to an older divergence as is seen in the eastern populations (YDS, WYS, LXS1 and LXS2). Conversely, the north–south orientation of the Nanling Mts., allowing various elevational shifts of plant species, can facilitate gradual retreat from north to south and short‐distance migrations during glacial and interglacial periods, thus promoting population diversification, larger population size and younger divergence as is seen in the populations of the Nanling Mts. (NLE1 and NLE2, and NLW1 through NLW5). The Nanling Mts. form a geographical boundary between the south‐ and the mid‐subtropical regions and possess complex topography and diverse habitats favoring population diversification. The orientation and physiography of the mountain ranges appear to have critically contributed to the geographical pattern of genetic variation between the eastern and western populations of *C*. *chuniana*.

### 4.3 Postglacial demographic expansion from the Nanling Mts. and secondary contact

FSC2 analyses yield a best‐fit model of isolation followed by demographic expansion and secondary contact (Table S4, Figure 6). Demographic expansion in the Nanling Mts. was inferred with notably increased effective population size (Table S4, Figure 6), indicating high local population diversification as is seen in Figure 3. The Nanling Mts., which are composed of five distinct ridges, has a long history of subtropical evergreen broadleaved forest (STEBF) in southern China (Fan et al., 2018; Xu et al., 2017). Its vegetation is characterized by highly varied elevational or longitudinal shifts, varying aspects of slope directions, high heterogeneity of soils, and abundant microhabitats (Huang et al., 2012; Qiu et al., 2011; Shen et al., 2019; Tang et al., 2006; Zhu et al., 2017), which together served as a buffer from climatic change and thus helped to confer relatively stable ecological conditions to these mountains during glacial periods. The Nanling Mts. are suggested to never have been glaciated and have maintained a nearly constant level of annual precipitation during the last glacial period as current (Xiao et al., 2007), making it more suitable for *C*. *chuniana* than other regions of subtropical China. Therefore, complex physiography plus long‐term stable ecological conditions in the Nanling Mts. across glacial cycles are thought to have preserved population genetic diversity, ultimately resulting in population size increase and opportunity for demographic expansion. Similar cases have been documented in widespread species in subtropical China, such as *Eurycorymbus cavaleriei*, *Loropetalum chinense*, and *Eomecon chionantha* (Gong et al., 2016; Tian et al., 2018; Wang et al., 2009).

The estimated time of secondary contact from our analysis (0.10 Ma) coincides with the Lushan–Tali Interglacial period in China (0.10–0.20 Ma; Duan et al., 1980), when a continuous geographical distribution of *C*. *chuniana* along the mountain ranges in subtropical China was detected by ecological niche modeling (ENM; Figure 2a). Because the Lushan–Tali Interglacial period somewhat overlaps with the last interglacial period (LIG; 0.12~0.14 Ma), its climate and environment was similar to that of the last interglacial period in China, when temperature increased and was estimated to be even higher than the present (Duan et al., 1980; Zhu et al., 2004). This suggests that the secondary contact may have occurred during this warmer time. Moreover, it is thought that the East Asia summer monsoon intensified during that time (Liu et al., 2018; Meng et al., 2018; Wang et al., 1999, 2007, 2012), thus providing more suitable habitat, especially considering that *C*. *chuniana* is adapted to mesic environments and most influenced by precipitation (Table S1).

Subtropical China has been long known as an area preserving higher species diversity than other regions of the Northern Hemisphere (Qian et al., 2005; Xiang et al., 2004). Such regional diversity bias is thought to be attributable to the high physiographical heterogeneity and diverse climate in the montane regions of subtropical China, which are advantageous for population colonization accompanied by repeated coalescence of populations through glacial cycles and postglacial increase. Our data may provide an explanation for higher species diversity of *Cercis* in subtropical China relative to any other part of its range in the Northern Hemisphere.

Additionally, our FSC2 analysis indicated bidirectional migrations occurring after the divergence of populations between the Nanling (NL) and eastern (ES) mountains, with the migration rate *M*_NL‐ES_ (2.13) higher than *M*_ES‐NL_ (0.33; Table S3, Figure 6). The migrations in *C*. *chuniana* appear to have proceeded primarily from the Nanling Mts. to the east. Many examples of plant species in East Asia exhibit a similar distribution pattern and migration route, such as *Tetrastigma hemsleyanum* and *Eomecon chionantha* (Tian et al., 2018; Wang, 1992a, 1992b; Wang et al., 2015). The question arises as to why the direction of contemporary migration is inferred from the Nanling Mts. toward the east, whereas the populations from the Nanling Mts. diverged more recently than those to the east. The Nanling Mts., with distinct phytogeography and long‐term stable ecological condition, are thought to be one of the glacial refugia for *C*. *chuniana*. Populations of *C*. *chuniana* are present at relatively higher elevations in the Nanling Mts. (>600 m) than the eastern ones (from 264–727 m). Seeds of *Cercis* are supposedly dispersed primarily by wind during the fall and winter (Dickson, 1990; Robertson, 1976). The mountains’ close proximity to each other may have facilitated west‐to‐east migration when the wind periodically blows most of the fruits from the branches straight across to the next mountain ranges from higher elevations to lower ones via closely adjacent stepping‐stone areas.

## 5 CONCLUSIONS

We aimed to advance understanding of the roles of mountain ranges and glacial cycles on the geographical distribution pattern of genetic variation for the plant species within the subtropical evergreen broadleaved forest (STEBF) in southern China. The orientation and physiography of the mountain ranges and the climate fluctuations across glacial cycles in this region appear to correlate with the geographical pattern of genetic variation in *C. chuniana*. The Nanling Mts. are considered an important glacial refugium for the preservation of genetic diversity during the glacial periods because of its complex physiography and long‐term stable ecological conditions. Our study provides molecular evidence on how topography and climate change affect the phylogeographic history of the representative species within STEBF of southern China. Study of additional plant groups with similar geographical distribution patterns is further required to assess whether the patterns from *Cercis* observed here apply more generally to the evolutionary history and past vegetation changes in the STEBF associated with physiography and climate fluctuation.

## CONFLICT OF INTEREST

The authors declare no conflict of interest to this work.

## Supporting information

Fig S1

Fig S2

Fig S3

Table S1‐S5

## Data Availability

The vcf files containing SNPs and the model scripts of this study are openly available in Figshare at http://dx.doi.org/10.6084/m9.figshare.15283395 and http://dx.doi.org/10.6084/m9.figshare.15097872, respectively.
