# Supplementary figures and images for: Population dynamics linked to glacial cycles in Cercis chuniana F. P. Metcalf (Fabaceae) endemic to the montane regions of subtropical China
Source: Evol Appl. 2021 Oct 7;14(11):2647–63. doi: 10.1111/eva.13301 (PMC8591333; doi:10.1111/eva.13301)

0.2 substitutions/site

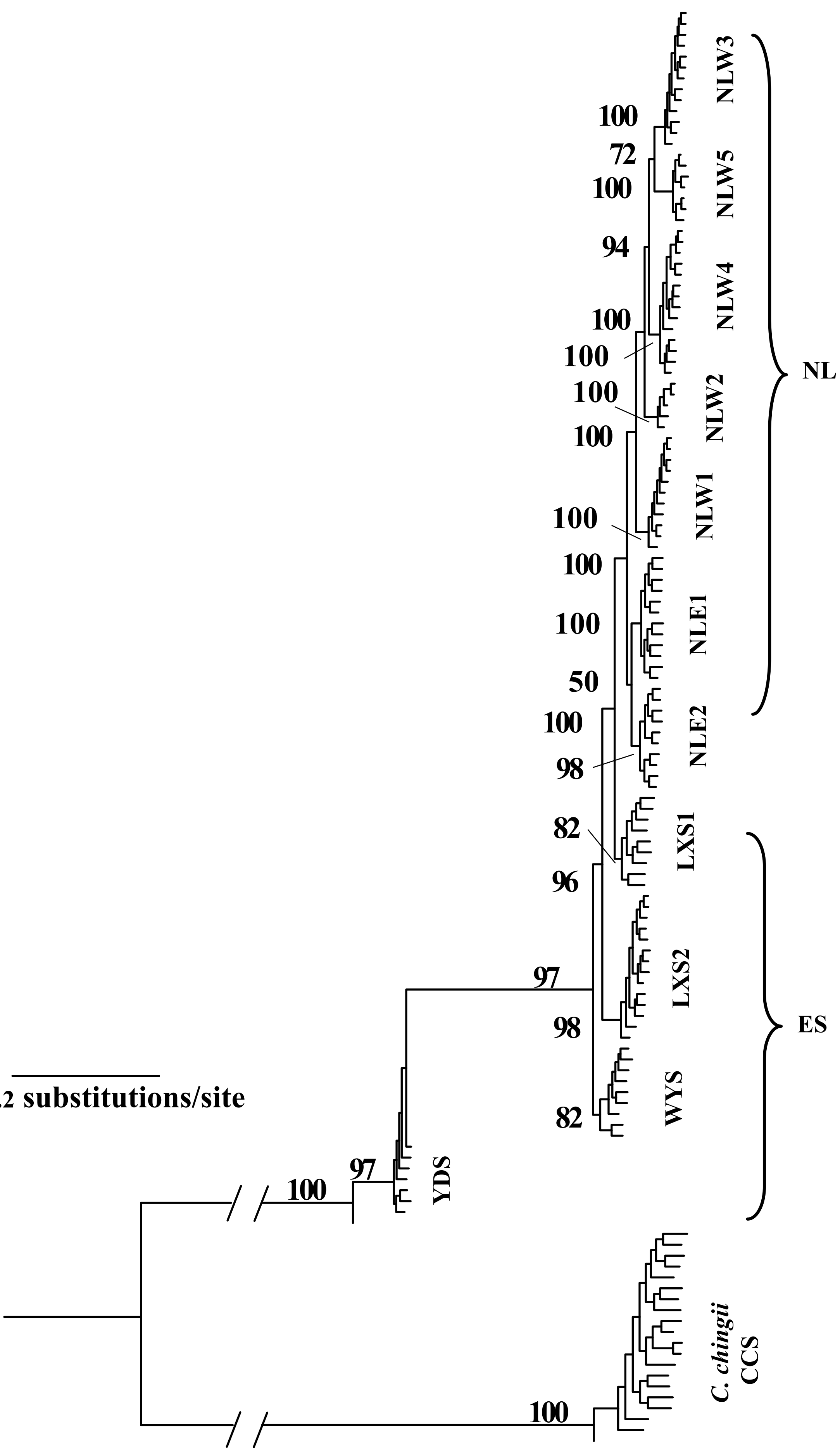

Supplement: Supplementary file 1 — Fig S1 [file EVA-14-2647-s001.pdf]

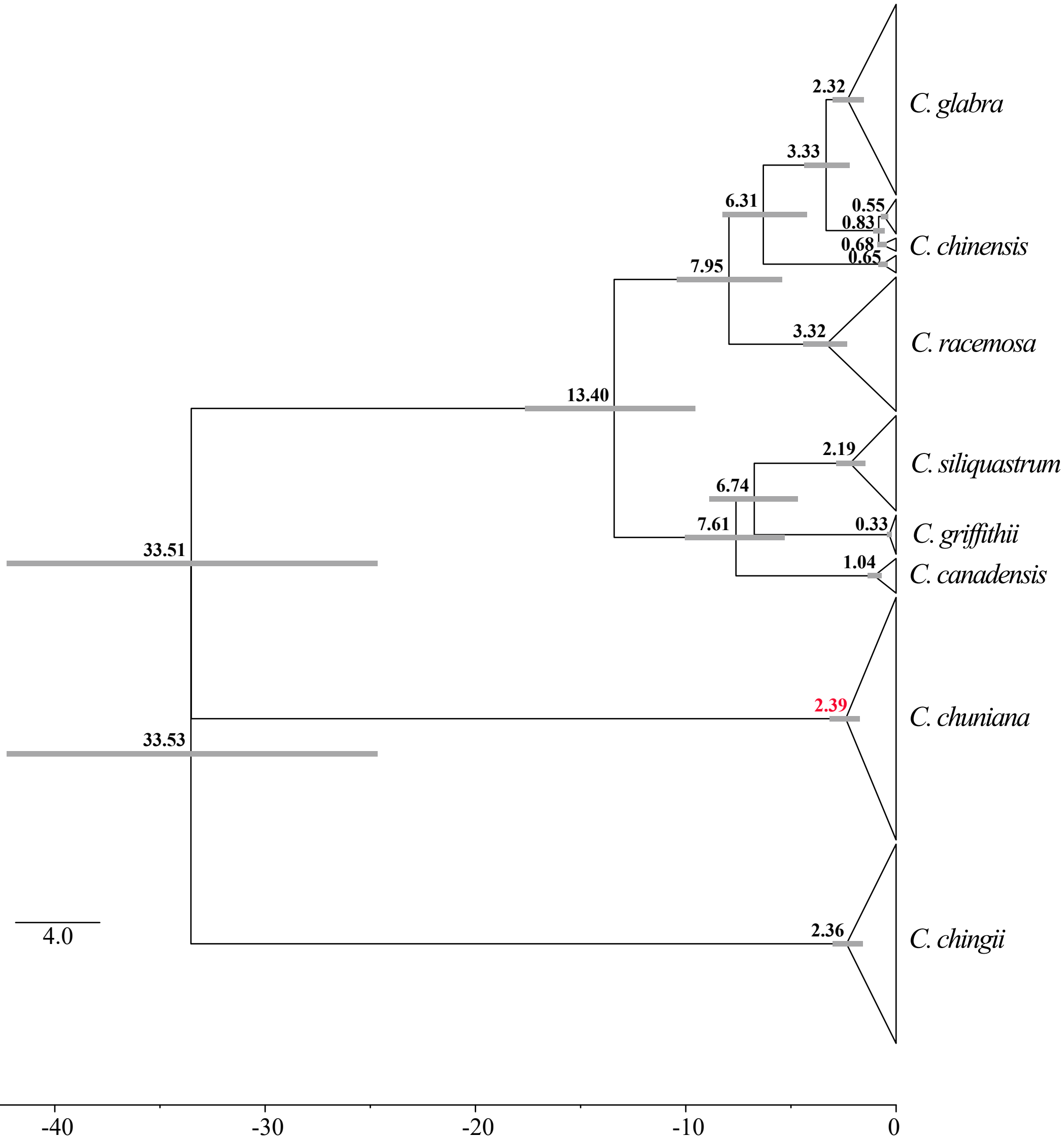

Supplement: Supplementary file 2 — Fig S2 [file EVA-14-2647-s004.pdf]

**a. NIS**

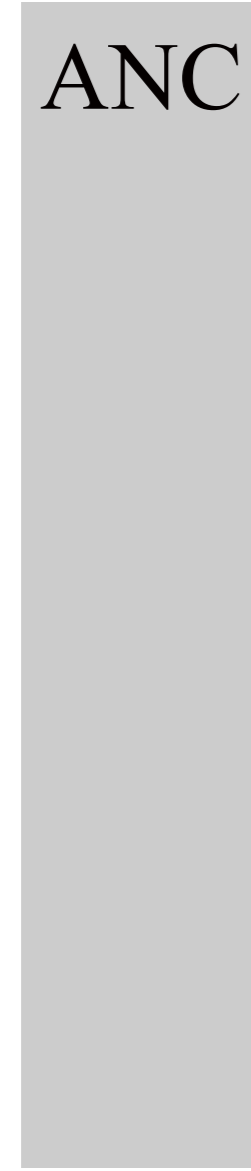

**b. IS**

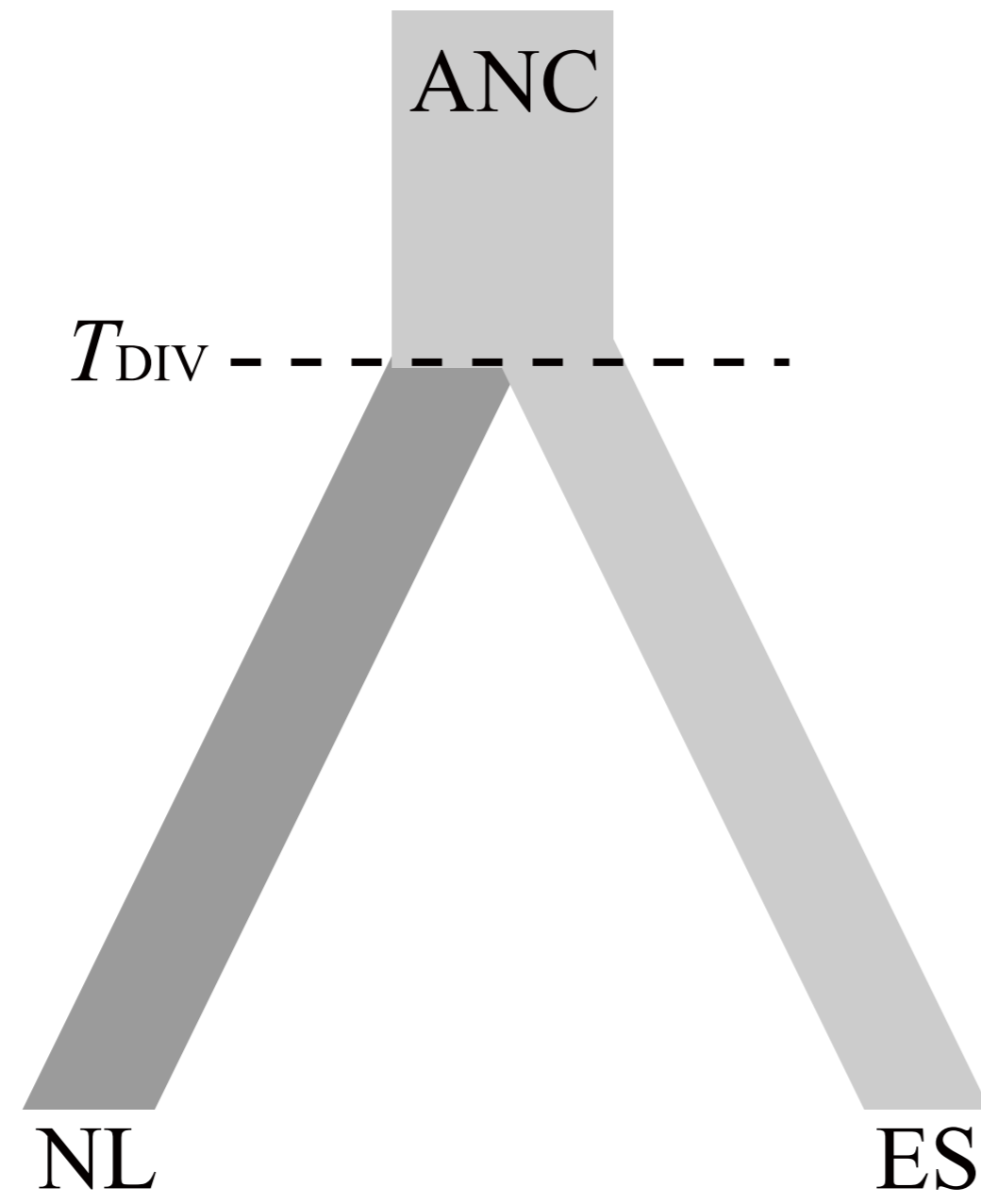

**c. IBOT**

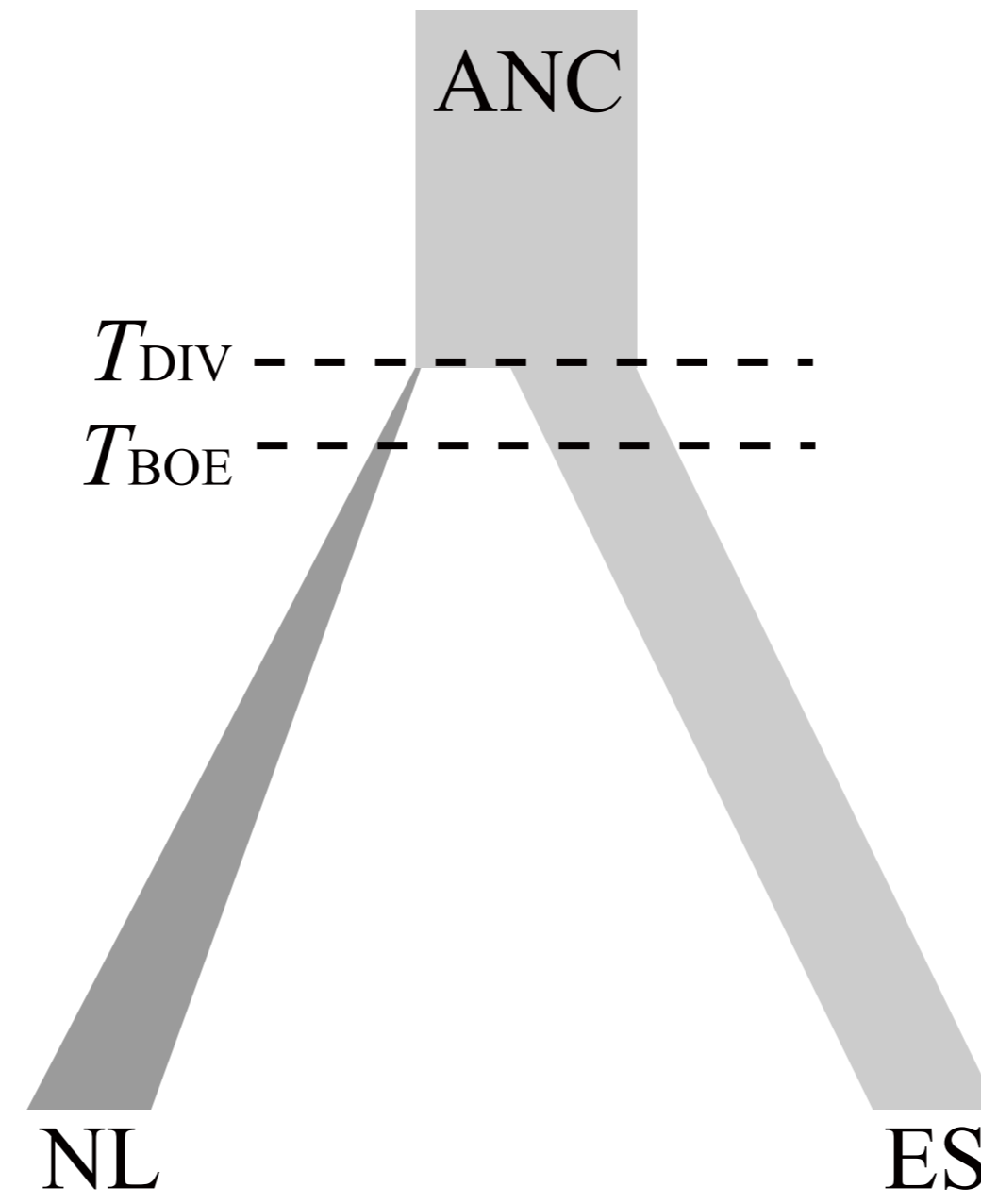

**d. IM**

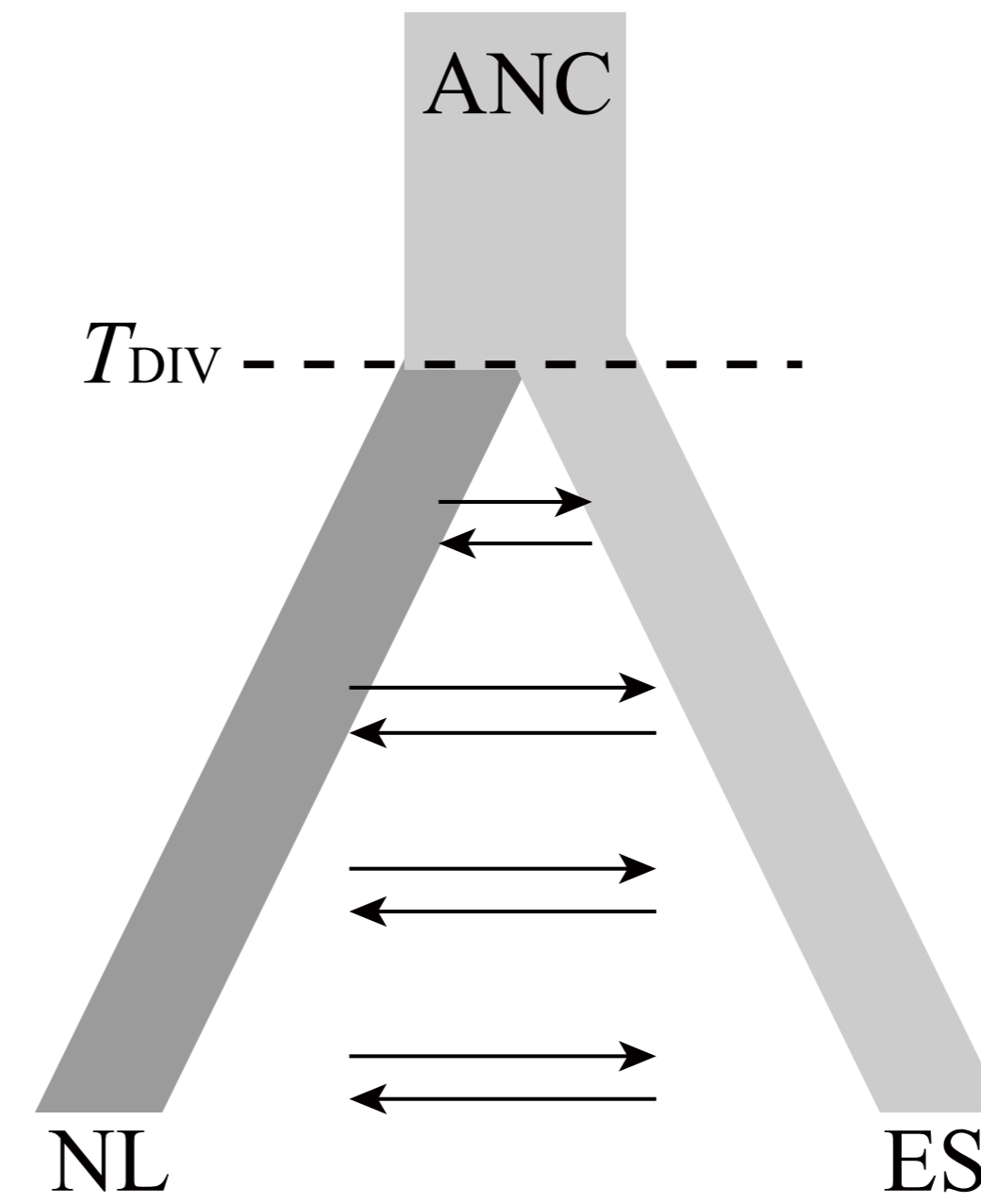

**e. IMEXP**

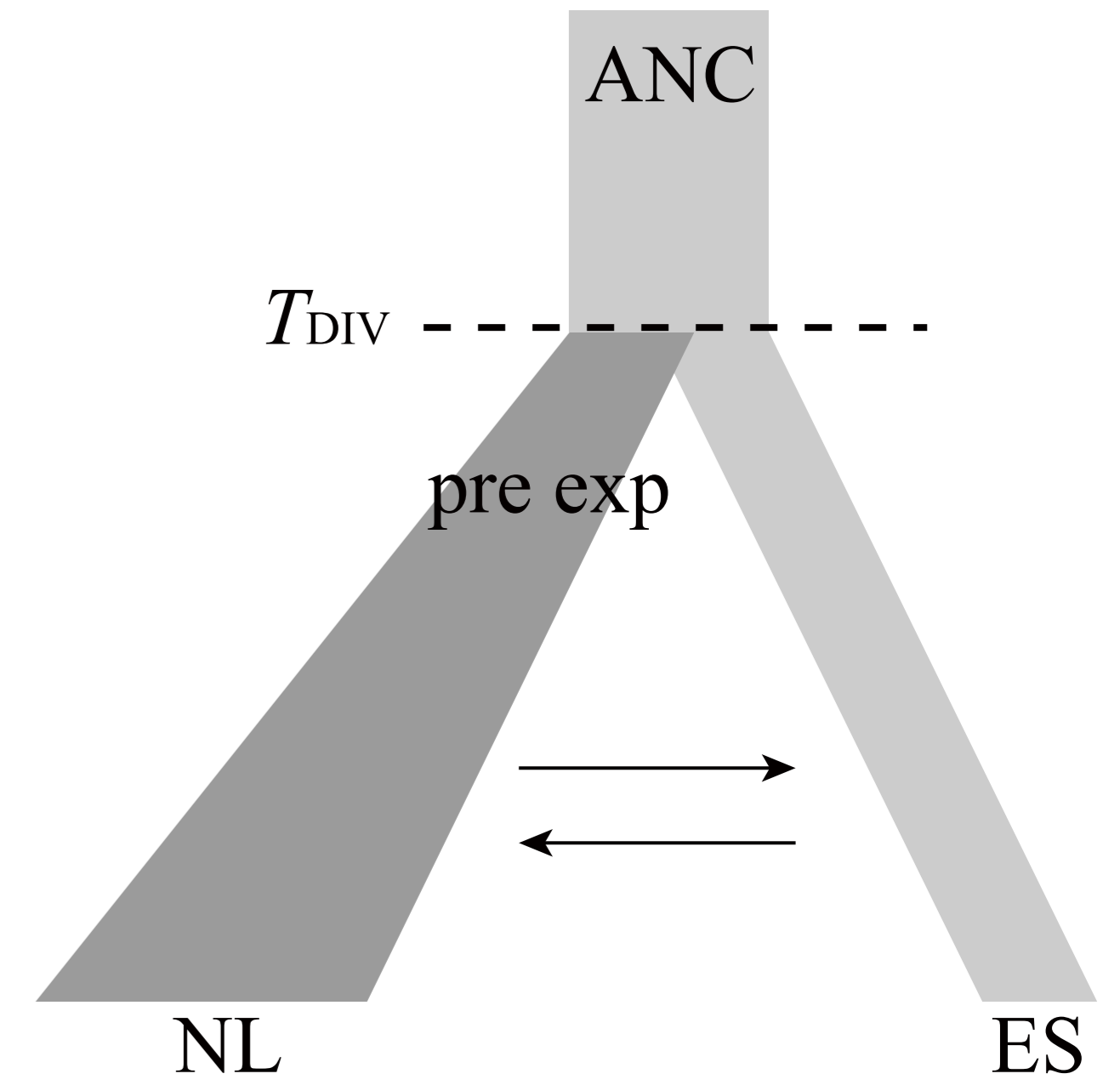

**f. SEC**

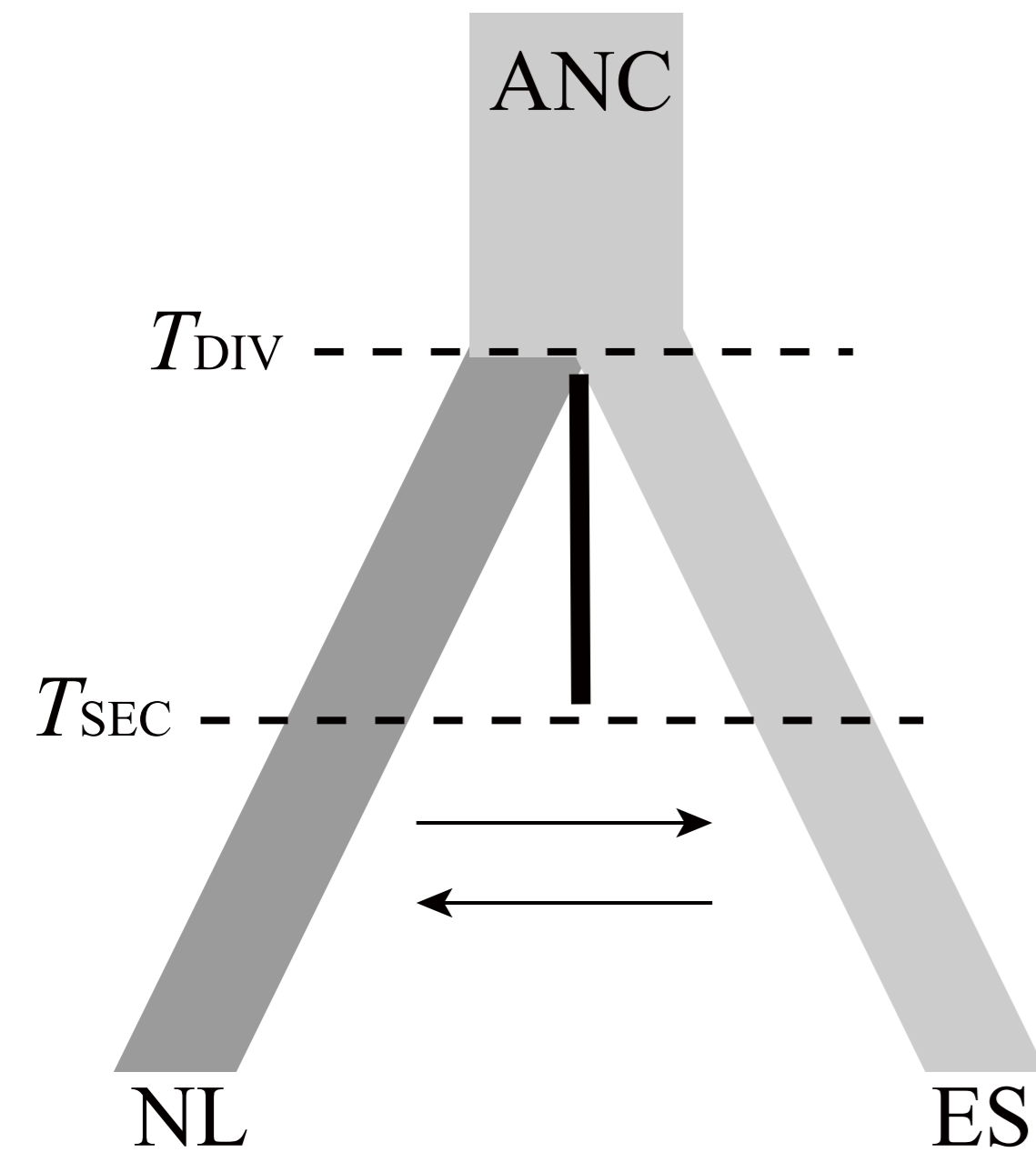

**g. SECEXP**

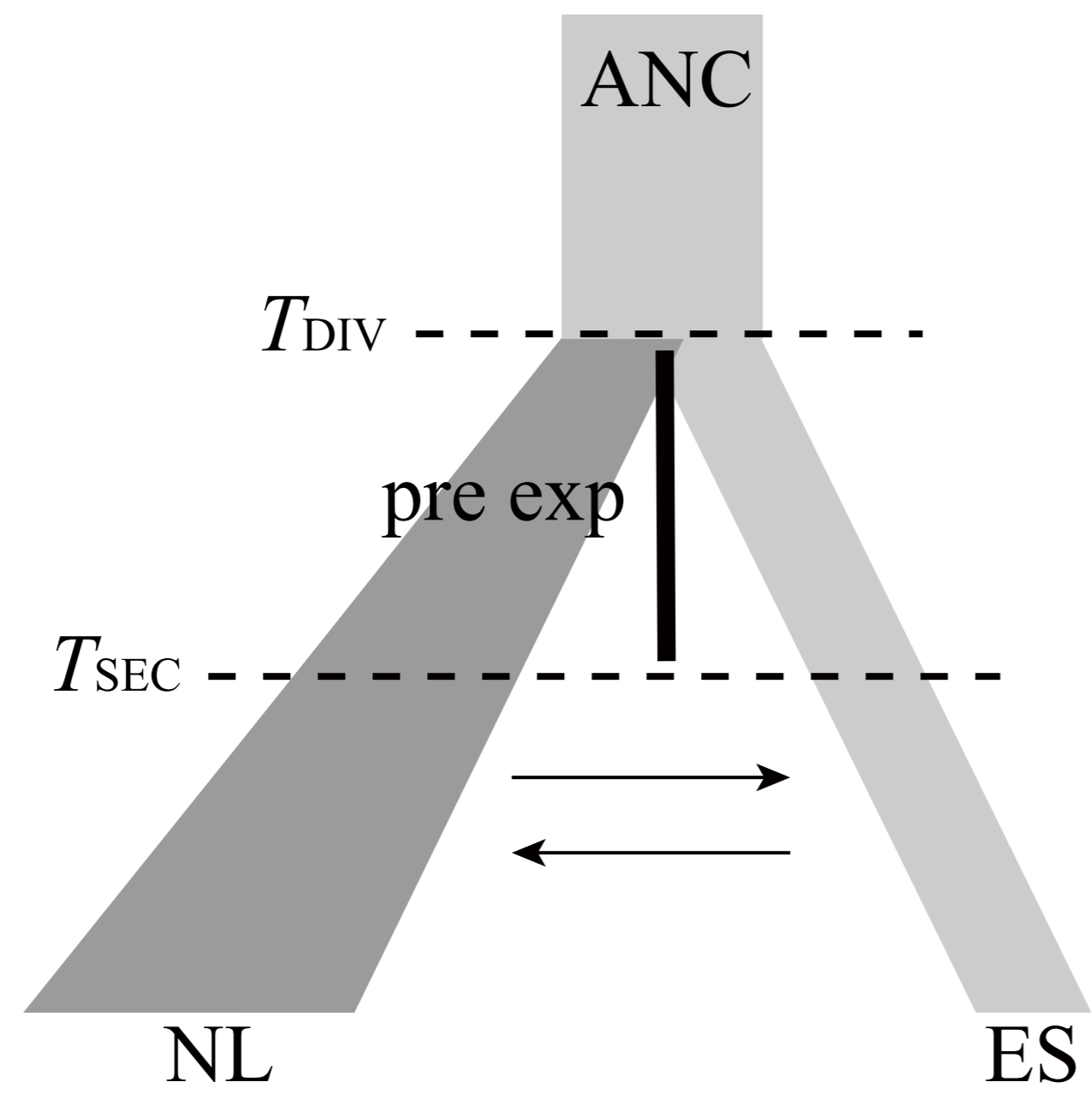

**h. IMone**

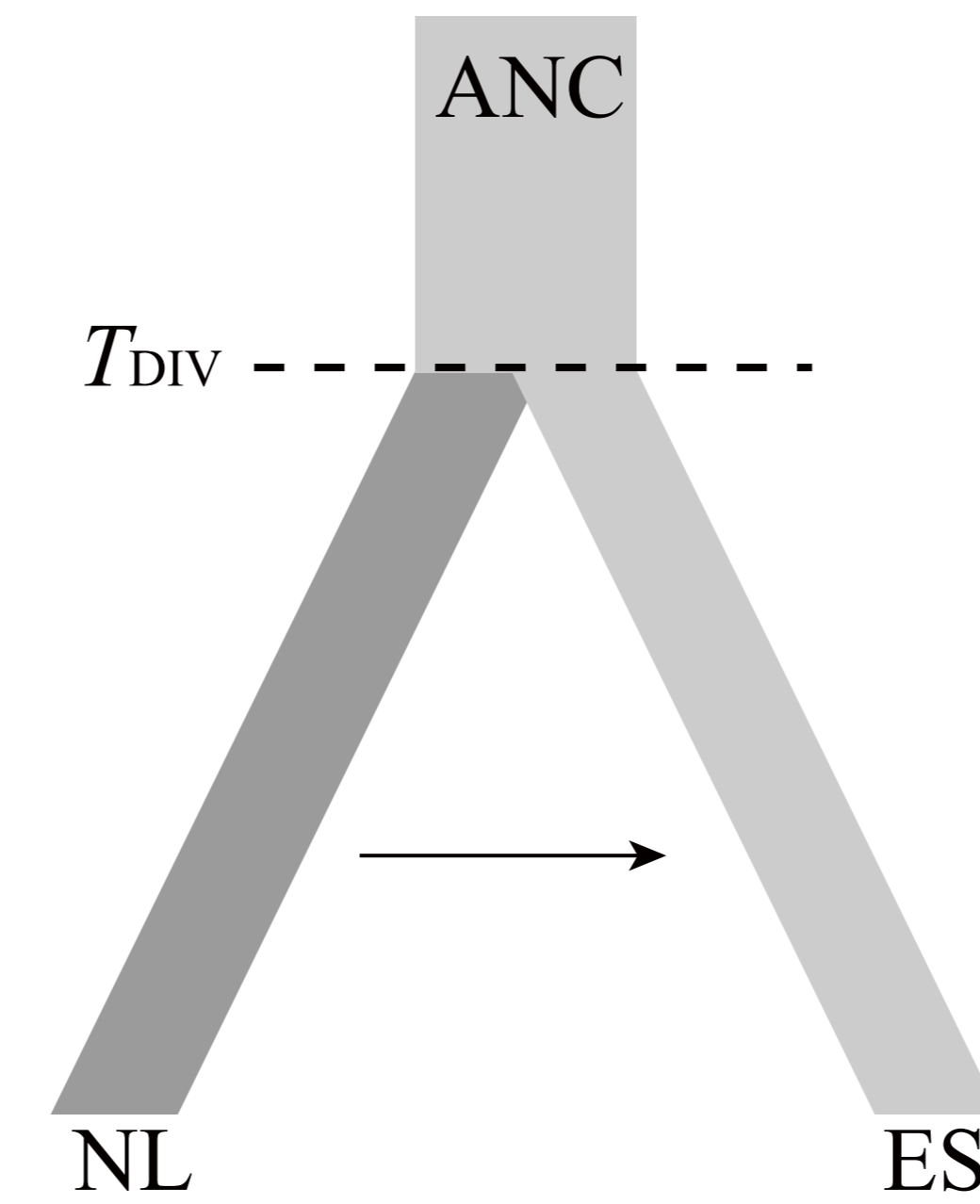

**i. IARM**

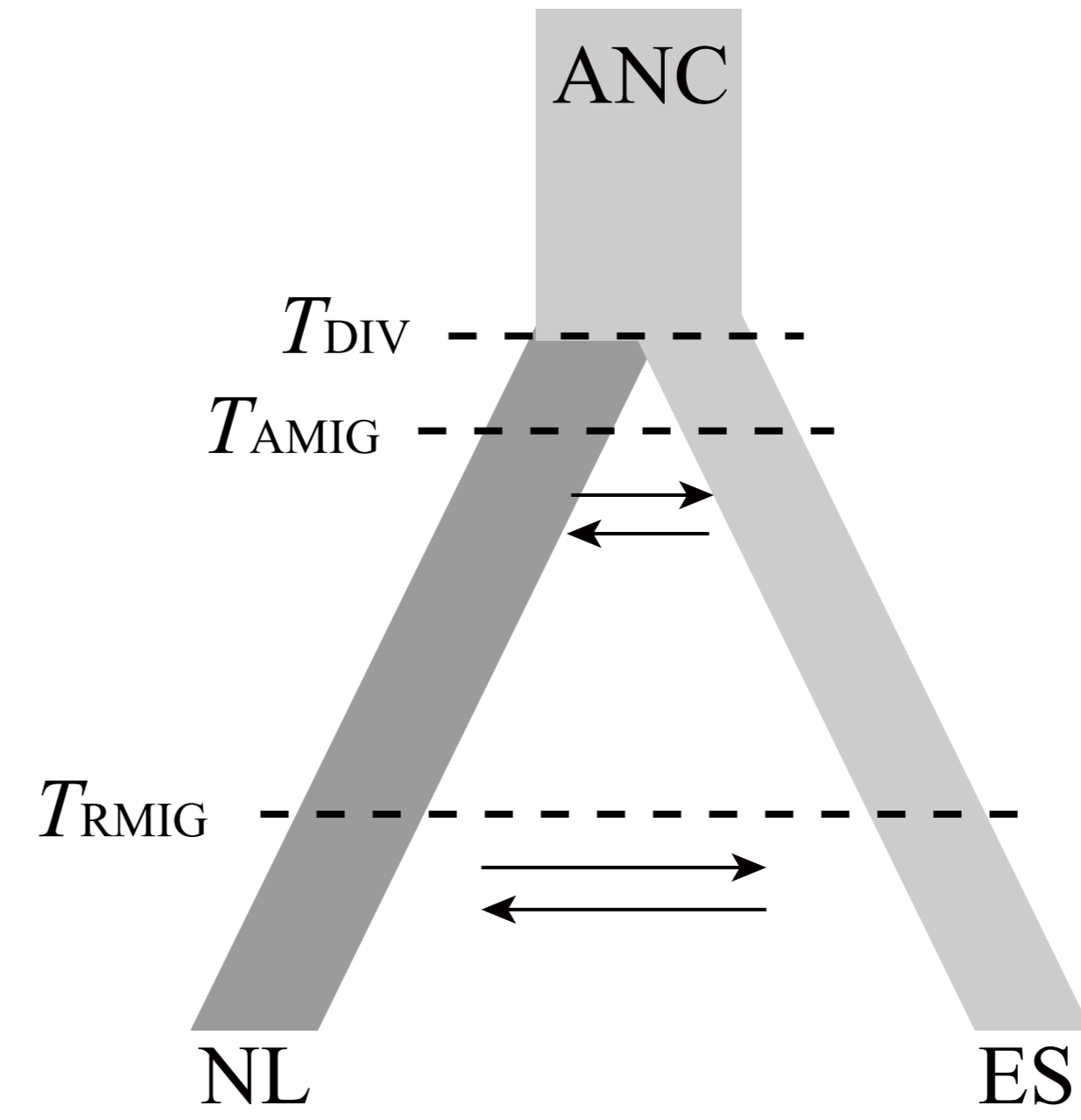

**j. IARMBOT**

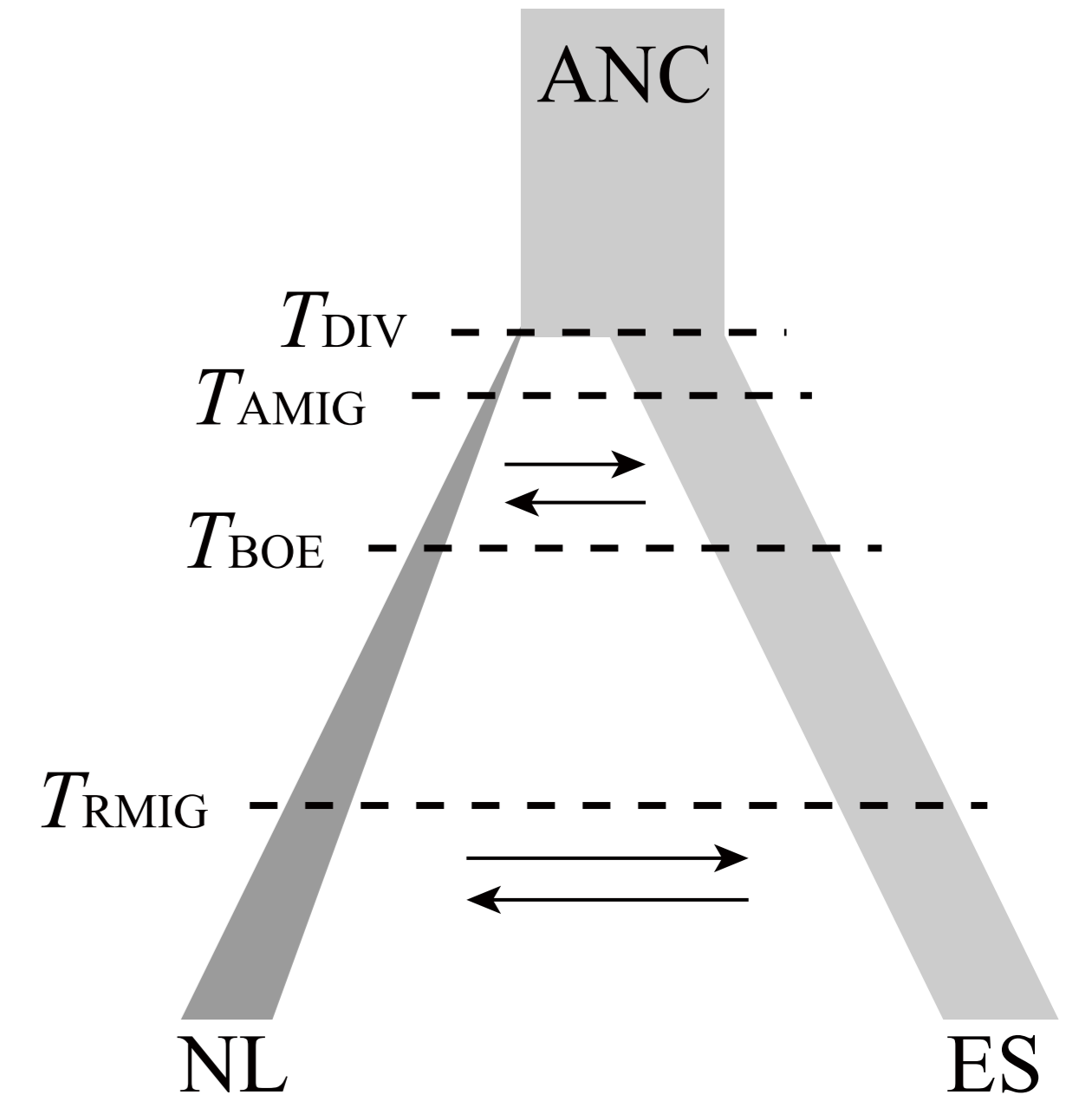

Supplement: Supplementary file 3 — Fig S3 [file EVA-14-2647-s002.pdf]
