# Supplementary material for: Population dynamics linked to glacial cycles in Cercis chuniana F. P. Metcalf (Fabaceae) endemic to the montane regions of subtropical China
Source: Evol Appl. 2021 Oct 7;14(11):2647–63. doi: 10.1111/eva.13301 (PMC8591333; doi:10.1111/eva.13301)
Supplement: Supplementary file 4 — Table S1‐S5 [file EVA-14-2647-s003.docx]

**TABLE S1** Top five Jackknife values of training gain and percentile contribution of environment variables for *Cercis chuniana* in ecological niche modeling

|  | 1 | 2 | 3 | 4 | 5 | Irreplaceable factor |
| --- | --- | --- | --- | --- | --- | --- |
| Jackknife of training gain | pr5 (2.6855) | pr6 (2.6106) | pr4 (2.2798) | tx2 (2.2736) | tx1 (2.2571) | pr12 |
| Percentile contribution | pr5 (52.8593) | bi4 (23.6844) | pr4 (6.4496) | pr6 (4.5167) | pr2 (4.3124) |  |

Abbreviations for parameters: pr, average precipitation (mm); bi, temperature seasonality (standard deviation ×100); tx, average maximum temperature (°C ×10). Numbers following parameters correspond to different months, e.g. 2 is February and 6 is June. “Irreplaceable factor” refers to the environmental variable that decreases the gain maximally if it is omitted. Therefore, it appears to be the most informative variable that cannot be replaced with the other variables.

**TABLE S2** The demographic models investigated in this study

| Model | Description |
| --- | --- |
| NIS | Model without isolation |
| IS | Isolation without migration after divergence |
| IBOT | Isolation without migration, but with bottleneck effect |
| IM | Isolation with migration, accompanied by bidirectional migration |
| IMEXP | Isolation with migration, accompanied by pre-expansion after divergence |
| SEC | Secondary contact with no immediate migration after divergence, but showing a recent migration and constant population size |
| SECEXP | Secondary contact with no immediate migration after divergence, but showing a recent migration and population expansion |
| IMone | Isolation with only one-way migration |
| IARM | Isolation with both ancient and recent migration after divergence |
| IARMBOT | Isolation with both ancient and recent migration after divergence, accompanied by bottleneck effect before a recent period |

**TABLE S3** Results of ten demographic models and 29 scenarios with parameters

| Model | Split Source | *Ne* | | | | |  | *M* | |  | *T* (Ma) | | | | |
| --- | --- | --- | --- | --- | --- | --- | --- | --- | --- | --- | --- | --- | --- | --- | --- |
|  |  | *Ne-*_ANC_ | *Ne-*_NL_ | *Ne-*_ES_ | *Ne-*_pre-exp_ | *Ne-*_BOE_ |  | *M*_NL-ES_ | *M*_ES-NL_ |  | *T*_DIV_ | *T*_SEC_ | *T*_BOE_ | *T*_AMIG_ | *T*_RMIG_ |
| NIS |  | 169,149 |  |  |  |  |  |  |  |  |  |  |  |  |  |
| IS | NL | 986,083 | 33,692 | 65,484 |  |  |  |  |  |  | 0.05 |  |  |  |  |
| IS | ES | 264,486 | 12,202 | 23,223 |  |  |  |  |  |  | 0.02 |  |  |  |  |
| IBOT_NL | NL | 9,529,130 | 53,924 | 288,605 |  | 16,366 |  |  |  |  | 0.07 |  | 0.02 |  |  |
| IBOT_NL | ES | 16,312,800 | 316,694 | 622,004 |  | 298,647 |  |  |  |  | 0.43 |  | 0.18 |  |  |
| IBOT_ES | ES | 9,701,310 | 449,401 | 827,004 |  | 627,978 |  |  |  |  | 0.59 |  | 0.26 |  |  |
| IBOT_ES | NL | 16,029,600 | 563,366 | 829,423 |  | 757,157 |  |  |  |  | 0.68 |  | 0.23 |  |  |
| IM | NL | 470,186 | 17,899 | 33,226 |  |  |  | 0.94 | 0.12 |  | 0.03 |  |  |  |  |
| IM | ES | 684,860 | 31,101 | 60,755 |  |  |  | 0.68 | 0.10 |  | 0.05 |  |  |  |  |
| IMEXP_NL | ES | 1,134,696 | 11,084 | 18,461 | 417 |  |  | 0.65 | 0.21 |  | 0.02 |  |  |  |  |
| IMEXP_NL | NL | 1,086,666 | 11,002 | 16,093 | 1,335 |  |  | 1.04 | 0.08 |  | 0.02 |  |  |  |  |
| IMEXP_ES | NL | 817,722 | 12,042 | 17,943 | 2,797 |  |  | 1.07 | 0.08 |  | 0.02 |  |  |  |  |
| IMEXP_ES | ES | 797,322 | 12,385 | 23,553 | 501 |  |  | 0.62 | 0.07 |  | 0.02 |  |  |  |  |
| SEC | NL | 846,889 | 52,212 | 13,971 |  |  |  | 2.7 | 0.35 |  | 1.38 | 0.06 |  |  |  |
| SEC | ES | 1,121,045 | 27,967 | 11,320 |  |  |  | 2.29 | 0.28 |  | 2.36 | 0.06 |  |  |  |
| SECEXP_NL | ES | 769,625 | 32,115 | 12,996 | 206 |  |  | 2.54 | 0.32 |  | 0.87 | 0.06 |  |  |  |
| **SECEXP_NL** | **NL** | **755,955** | **57,495** | **14,955** | **866** |  |  | **2.13** | **0.33** |  | **1.60** | **0.10** |  |  |  |
| SECEXP_ES | NL | 828,421 | 37,431 | 11,828 | 546 |  |  | 2.34 | 0.34 |  | 1.64 | 0.06 |  |  |  |
| SECEXP_ES | ES | 193,818 | 34,118 | 11,880 | 1,937 |  |  | 2.22 | 0.31 |  | 2.90 | 0.05 |  |  |  |
| IMone_NE | NL | 939,333 | 11,549 | 17,562 |  |  |  | 1.15 |  |  | 0.02 |  |  |  |  |
| IMone_NE | ES | 1,090,042 | 11,306 | 16,922 |  |  |  | 0.86 |  |  | 0.02 |  |  |  |  |
| IMone_EN | NL | 791,776 | 11,147 | 22,923 |  |  |  |  | 0.07 |  | 0.02 |  |  |  |  |
| IMone_EN | ES | 658,658 | 11,895 | 26,139 |  |  |  |  | 0.18 |  | 0.02 |  |  |  |  |
| IARM | ES | 192,680 | 57,814 | 21,680 |  |  |  | 2.43 (Ancient) | 0.26 (Ancient) |  | 3.09 |  |  | 2.83 | 0.08 |
|  |  |  |  |  |  |  |  | 2.43 (Recent) | 0.26 (Recent) |  |  |  |  |  |  |
| IARM | NL | 618,514 | 70,997 | 17,843 |  |  |  | 2.59 (Ancient) | 0.29 (Ancient) |  | 1.86 |  |  | 1.77 | 0.07 |
|  |  |  |  |  |  |  |  | 2.59 (Recent) | 0.29 (Recent) |  |  |  |  |  |  |
| IARMBOT_NL | NL | 70,634 | 41,594 | 11,510 |  | 5,578 |  | 0.3 (Ancient) | 0.33 (Ancient) |  | 2.15 |  | 0.84 | 2.12 | 0.05 |
|  |  |  |  |  |  |  |  | 2.2 (Recent) | 0.33 (Recent) |  |  |  |  |  |  |
| IARMBOT_NL | ES | 76,705 | 32,669 | 13,462 |  | 1,593 |  | 0.13 (Ancient) | 0.34 (Ancient) |  | 2.70 |  | 0.96 | 2.60 | 0.05 |
|  |  |  |  |  |  |  |  | 2.59 (Recent) | 0.34 (Recent) |  |  |  |  |  |  |
| IARMBOT_ES | ES | 174,951 | 41,341 | 16,371 |  | 1,612 |  | 2.79 (Ancient) | 0.01 (Ancient) |  | 2.50 |  | 1.00 | 2.35 | 0.06 |
|  |  |  |  |  |  |  |  | 2.79 (Recent) | 0.26 (Recent) |  |  |  |  |  |  |
| IARMBOT_ES | NL | 4,598 | 26,715 | 11,733 |  | 2,813 |  | 2.74 (Ancient) | 0.03 (Ancient) |  | 2.99 |  | 1.17 | 2.83 | 0.04 |
|  |  |  |  |  |  |  |  | 2.74 (Recent) | 0. 3(Recent) |  |  |  |  |  |  |

NL, populations in the Nanling Mts.; ES, eastern populations. *Ne*, effective population size in number of haploid genomes of the ancestral populations (*Ne_ANC_*), the populations in Nanling Mts. (*Ne_NL_*), the eastern populations (*Ne_ES_*) before expansion (*Ne_pre-exp_*) or during bottleneck effect (*Ne-_BOE_*). *M*, migration rate in numbers of individuals between populations with direction indicated as *M_NL-ES_* or *M_ES-NL_*. *T*, time in millions of years ago (Ma) based on the equation *T* = the number of generations × life time (years), i.e., 5 years for *C. chuniana*. *T* is estimated for population divergence (*T_DIV_*), bottleneck event (*T_BOE_*), and secondary contact (*T*_SEC_) as well as the time in generations for migration (*T*_MIG_), and ancient migration (*T_A_*_MIG_) or recent migration (*T*_RMIG_). The best fit model is in boldface. The demographic model descriptions follow the abbreviations in Table S2.

**TABLE S4** The likelihood (ΔLhood) of each scenario for each demographic model investigated with FSC2 used for NL versus ES groups, showing their respective Akaike information criteria (AIC) scores and ΔAIC scores.

| Demographical Model | Split Source | ΔLhood | AIC | ΔAIC |
| --- | --- | --- | --- | --- |
| NIS |  | 3,245.574 | 118,788.871 | 22958.9316 |
| IS | NL | 1,529.496 | 99,107.38621 | 3277.4468 |
| IS | ES | 1,486.504 | 98,909.40073 | 3079.4613 |
| IBOT_NL | NL | 1,511.412 | 98,755.68747 | 2925.7481 |
| IBOT_NL | ES | 1,497.578 | 98,691.97954 | 2862.0401 |
| IBOT_ES | ES | 1,506.201 | 99,004.10877 | 3174.1694 |
| IBOT_ES | NL | 1,532.677 | 99,126.03525 | 3296.0958 |
| IM | NL | 1,450.55 | 98,747.82644 | 2917.887 |
| IM | ES | 1,432.129 | 98,662.9946 | 2833.0552 |
| IMEXP_NL | ES | 1,384.088 | 98,171.64732 | 2341.7079 |
| IMEXP_NL | NL | 1,397.425 | 98,233.06648 | 2403.1271 |
| IMEXP_ES | NL | 1,389.049 | 98,466.91241 | 2636.973 |
| IMEXP_ES | ES | 1,385.08 | 98,448.63449 | 2618.6951 |
| SEC | NL | 950.122 | 96,447.27033 | 617.3309 |
| SEC | ES | 985.905 | 96,612.05714 | 782.1177 |
| SECEXP_NL | ES | 973.308 | 96,283.93551 | 453.9961 |
| **SECEXP_NL** | **NL** | **874.724** | **95,829.93942** | 0 |
| SECEXP_ES | NL | 956.69 | 96,479.82564 | 649.8862 |
| SECEXP_ES | ES | 987.915 | 96,623.62208 | 793.6827 |
| IMone_NE | NL | 1,407.77 | 98,548.81726 | 2718.8778 |
| IMone_NE | ES | 1,404.472 | 98,533.62941 | 2703.69 |
| IMone_EN | NL | 1,450.23 | 98,744.35279 | 2914.4134 |
| IMone_EN | ES | 1,426.434 | 98,634.76816 | 2804.8287 |
| IARM | ES | 1,106.221 | 97,166.13279 | 1336.1934 |
| IARM | NL | 1,066.316 | 96,982.36348 | 1152.4241 |
| IARMBOT_NL | NL | 1,007.379 | 96,442.52972 | 612.5903 |
| IARMBOT_NL | ES | 1,054.003 | 96,657.24118 | 827.3018 |
| IARMBOT_ES | ES | 1,100.852 | 97,145.40764 | 1315.4682 |
| IARMBOT_ES | NL | 1,049.823 | 96,910.41041 | 1080.471 |

NL, populations in the Nanling Mts.; ES, eastern populations. ΔLhood, difference in likelihood between the maximum possible and that obtained from the simulations in log_10_ units; AIC, Akaike Information Criterion; ΔAIC, difference in AIC and that of the best model. The best fit model is in boldface.

TABLE S5 Pairwise *Fst* values among populations of *Cercis chuniana* and one population fo *C. chingii* (CCS).

|  | YDS | WYS | LXS1 | LXS2 | NLE1 | NLE2 | NLW1 | NLW2 | NLW3 | NLW4 | NLW5 | CCS |
| --- | --- | --- | --- | --- | --- | --- | --- | --- | --- | --- | --- | --- |
| YDS | / |  |  |  |  |  |  |  |  |  |  |  |
| WYS | 0.47 |  |  |  |  |  |  |  |  |  |  |  |
| LXS1 | 0.45 | 0.16 |  |  |  |  |  |  |  |  |  |  |
| LXS2 | 0.51 | 0.21 | 0.18 |  |  |  |  |  |  |  |  |  |
| NLE1 | 0.46 | 0.20 | 0.11 | 0.22 |  |  |  |  |  |  |  |  |
| NLE2 | 0.46 | 0.20 | 0.11 | 0.21 | 0.09 |  |  |  |  |  |  |  |
| NLW1 | 0.54 | 0.28 | 0.18 | 0.29 | 0.17 | 0.16 |  |  |  |  |  |  |
| NLW2 | 0.57 | 0.29 | 0.18 | 0.30 | 0.17 | 0.17 | 0.23 |  |  |  |  |  |
| NLW3 | 0.55 | 0.29 | 0.20 | 0.31 | 0.20 | 0.19 | 0.23 | 0.21 |  |  |  |  |
| NLW4 | 0.54 | 0.28 | 0.19 | 0.30 | 0.18 | 0.18 | 0.22 | 0.18 | 0.19 |  |  |  |
| NLW5 | 0.60 | 0.31 | 0.21 | 0.33 | 0.21 | 0.20 | 0.26 | 0.26 | 0.23 | 0.20 |  |  |
| CCS | 0.86 | 0.84 | 0.82 | 0.85 | 0.84 | 0.83 | 0.86 | 0.85 | 0.87 | 0.86 | 0.86 | / |

*Fst*: 0~0.05, low differentiation; 0.05~0.15, moderately differentiation; 0.15~0.25, high genetic differentiation; > 0.25, very high genetic differentiation.

**FIGURE LEGENDS**

**FIGURE S1 Phylogram of the best phylogenetic tree of *Cercis chuniana* populations based on maximum-likelihood (ML).** Bootstrap percentages (> 50) in the ML tree are indicated above the branches. NL refers to the populations in Nanling Mts., whereas ES refers to the populations in the east. All other abbreviations are population abbreviations from Table 1. *Cercis chingii* was used as the outgroup. The unit for the branch length is substitutions/site.

**FIGURE S2 Bayesian time estimation for the genus *Cercis*. Branch lengths were transformed via Markov chain Monte Carlo (MCMC) simulations in the Bayesian time estimation. The light grey bars indicate 95% confidence intervals. We used *C. chingii* as the outgroup. The crown age of *C. chuniana* is indicated in red.**

**FIGURE S3 Ten models used to elucidate demography in *Cercis chuniana*.** The first model is without isolation. Each of the remaining models is a two-population isolation and migration model that involves different scenarios. The models correspond to Table S2 and the free parameters are all given in Table S3. In each model, the top grey boxes represent the ancestral population. Time progresses from top to bottom (current time). Migration directions between the two groups are indicated with arrows. Vertical black bars represent a period of isolation of lineages before migration initiates at secondary contact. Demographic expansion is indicated by the increasing areas of the grey ladder shapes. Bottleneck effect is indicated by the decreasing area of the small grey ladder shapes. NL, populations in the Nanling Mts.; ES, eastern populations. *Ne*, effective population size in number of haploid genomes of the ancestral populations (*Ne_ANC_*), the populations in Nanling Mts. (*Ne_NL_*), the eastern populations (*Ne_ES_*), before expansion (*Ne_pre-exp_*), or during bottleneck effect (*Ne-_BOE_*). *M*, migration rate in numbers of individuals between populations with direction indicated as *M_NL-ES_* or *M_ES-NL_*. *T*, time in millions of years ago (Ma) based on the equation *T* = the number of generations × life time (years), i.e., 5 years for *C. chuniana*. *T* is estimated for population divergence (*T_DIV_*), bottleneck event (*T_BOE_*), and secondary contact (*T*_SEC_) as well as the time in generations for migration (*T*_MIG_), and ancient migration (*T_A_*_MIG_) or recent migration (*T*_RMIG_). The demographic model descriptions follow the abbreviations in Table S2.
